# Endosomal sorting drives the formation of axonal prion protein endoggresomes

**DOI:** 10.1126/sciadv.abg3693

**Published:** 2021-12-22

**Authors:** Romain Chassefeyre, Tai Chaiamarit, Adriaan Verhelle, Sammy Weiser Novak, Leonardo R. Andrade, André D. G. Leitão, Uri Manor, Sandra E. Encalada

**Affiliations:** 1 Department of Molecular Medicine, The Scripps Research Institute, La Jolla, CA 92037, USA.; 2 Dorris Neuroscience Center, The Scripps Research Institute, La Jolla, CA 92037, USA.; 3 Neurodegeneration New Medicines Center, The Scripps Research Institute, La Jolla, CA 92037, USA.; 4 Waitt Advanced Biophotonics Center, Salk Institute for Biological Studies, La Jolla, CA 92037, USA.

## Abstract

The pathogenic aggregation of misfolded prion protein (PrP) in axons underlies prion disease pathologies. The molecular mechanisms driving axonal misfolded PrP aggregate formation leading to neurotoxicity are unknown. We found that the small endolysosomal guanosine triphosphatase (GTPase) Arl8b recruits kinesin-1 and Vps41 (HOPS) onto endosomes carrying misfolded mutant PrP to promote their axonal entry and homotypic fusion toward aggregation inside enlarged endomembranes that we call endoggresomes. This axonal rapid endosomal sorting and transport-dependent aggregation (ARESTA) mechanism forms pathologic PrP endoggresomes that impair calcium dynamics and reduce neuronal viability. Inhibiting ARESTA diminishes endoggresome formation, rescues calcium influx, and prevents neuronal death. Our results identify ARESTA as a key pathway for the regulation of endoggresome formation and a new actionable antiaggregation target to ameliorate neuronal dysfunction in the prionopathies.

## Teaser

Mutant prion protein particles form axonal aggregates inside endolysosomes via axonal transport and endosomal fusion.

## INTRODUCTION

The progressive accumulation of misfolded proteins as intraneuronal pathologic aggregates is a feature of neurodegenerative disorders (*1*). Axons are particularly vulnerable to the formation of aggregates, which are often observed at dystrophic swelling sites, and can contain wild-type (WT) or mutant misfolded proteins that lose solubility and become cytotoxic to the neuron and to neighboring cells via spreading (*1*–*3*). The molecular mechanisms and pathways that drive the formation of misfolded protein aggregates versus their clearance in axons remain undefined.

To regulate sorting, signaling, and metabolic function, axons rely on active microtubule-based transport machinery that enables the distribution of vesicles and organelles to distal axonal domains via kinesin-dependent anterograde movement (*4*), and the retrograde movement of axonal cargos toward the soma by cytoplasmic dynein, for efficient lysosomal-based degradation (*5*). Axonal homeostasis also depends on an interconverting endolysosomal trafficking system that spreads over long distances. The regulation of intraneuronal endolysosomal sorting is, in part, orchestrated by Arl8b, an Arf-like (Arl) family of small guanosine triphosphatase (GTPase) that recruits kinesin, and the multiunit homotypic fusion and protein sorting (HOPS) complex onto late endosomes (LEs) to drive their motility to the cell periphery and toward homotypic fusion with other LEs (*6*, *7*). Axonal transport and endolysosomal trafficking allow the sorting of neuronal cargos between subcellular vesicular compartments in axons. Whether and how misfolded and aggregation-prone proteins co-opt axonal endolysosomal trafficking systems to form aggregates in axons are unknown.

Mutations in prion protein (PrP) *PRNP* gene associated with inherited human prion disease (*8*) can result in the intraneuronal deposition of insoluble PrP aggregates, including within dystrophic axons in brains of patients with prion disease, as well as in familial rodent models of the disease (*9*–*11*). The mechanisms of misfolded PrP aggregate formation and deposition within neurons, as well as their connection to neurotoxicity, remain debated. Mutant and infectious PrP have been observed to accumulate in the cytosol as aggresomes (*12*–*14*). However, misfolded PrP also largely accumulates inside endocytic membrane compartments in cultured cells and in neurons in human brains of patients with prion disease (*13*, *15*–*19*), pointing toward a possible role of endolysosomal pathways in PrP aggregation. Endolysosomal membrane sorting and trafficking are central to PrP physiology and pathology, as both PrP^C/WT^ (cellular/WT) and misfolded mutant PrP are glycosylphosphatidylinositol (GPI)–anchored proteins that transit to the cell surface from the endoplasmic reticulum (ER) via the Golgi in the lumen of membrane compartments, from where they are endocytosed back for recycling in endosomes, or that transit to lysosomes for degradation (*17*, *20*–*22*). PrP also resides in recycling endosomes or in multivesicular bodies (MVBs), locations critical for PrP conversion to misfolded conformations (*15*, *16*) and for its packaging for extracellular release in exosomes during cell-to-cell spread (*23*). How mutant PrP sorts between endolysosomal compartments in axons is unclear, but identification of the subcellular itinerary where mutant PrP aggregates form, transit, and become neurotoxic in axons is critical for understanding the basis of axonotoxicity in the prionopathies and may have implications for understanding the axonal etiology of other neurodegenerative disorders. Here, by systematically analyzing the neuronal trafficking itineraries of misfolded mutant PrP that causes familial prion disease in humans, we identified endosomal pathways that impose markedly different fates on mutant PrP: One pathway drives mutant PrP for degradation in the soma, while a second Arl8b/kinesin-1/HOPS axis propels mutant PrP into the axon for sorting, fusion, and aggregation inside endolysosomal structures that we term endoggresomes. Axonal endoggresomes impair calcium dynamics and reduce neuronal viability, but reducing the function of Arl8b/kinesin-1/HOPS components inhibits endoggresome formation in axons and circumvents neuronal toxicity and neuronal death. Our findings identify the endolysosomal trafficking system as a key player in the discriminating itinerary of misfolded mutant PrP within neurons toward degradation versus aggregation and point to the Arl8b/kinesin-1/HOPS pathway as an antiaggregation target amenable to therapeutic intervention that can modulate neuronal dysfunction in the prionopathies.

## RESULTS

### Mutant PrP is transported by kinesin-1 in vesicles and forms aggregates in axons

**Fig. 1.**
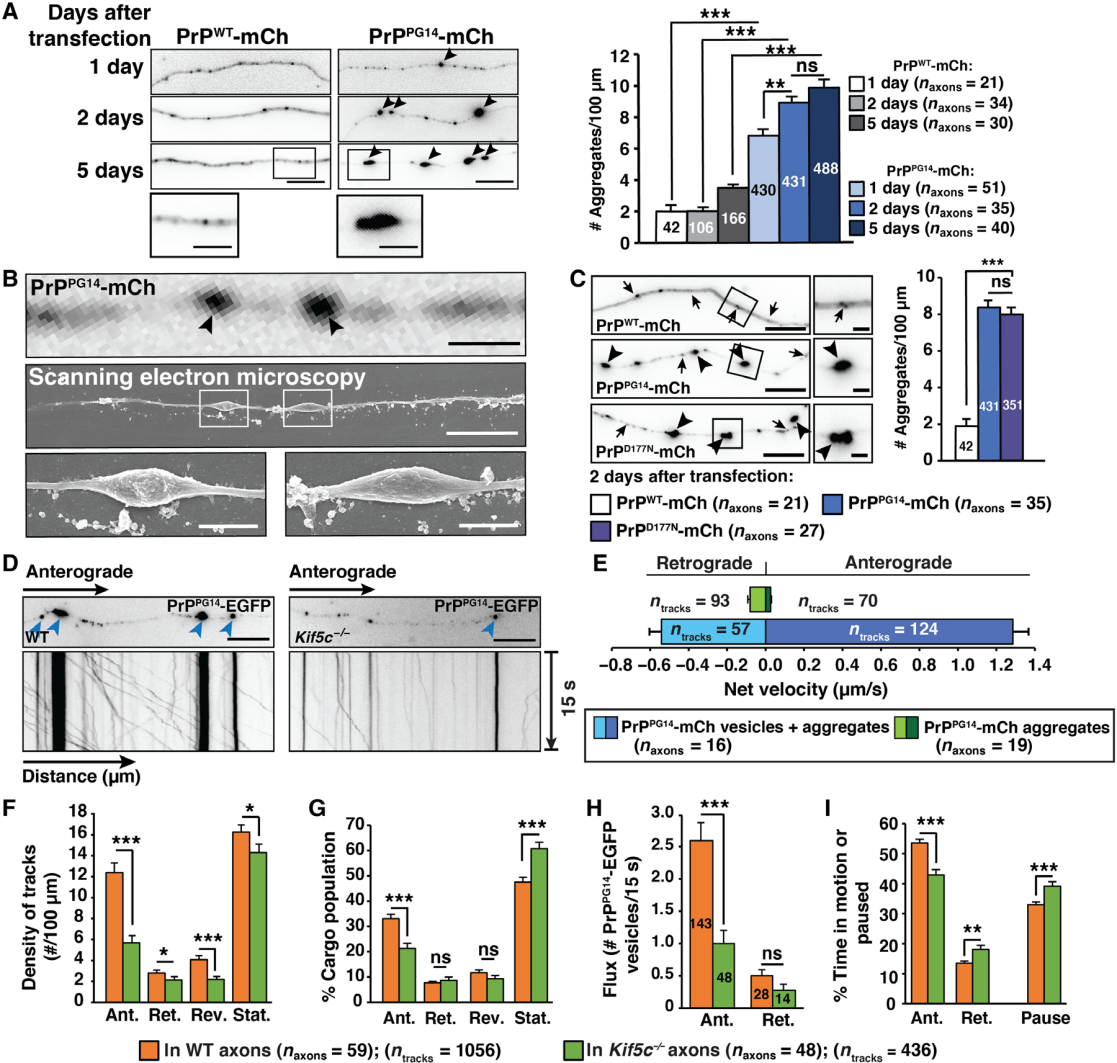
PrP^PG14^ forms intra-axonal aggregates. (**A**) Representative images of axons expressing PrP^WT^-mCh and PrP^PG14^-mCh (left). Arrowheads indicate aggregates in all images. Scale bars, 10 μm. Insets show enlargements. Scale bars, 5 μm. Aggregate densities (right). (**B**) Correlative light-electron microscopy images of swellings 2 days after PrP^PG14^-mCh transfection. Swelling number = 20. Scale bars, 10 μm. Insets show enlargements. Scale bars, 2 μm. (**C**) Images of axons expressing indicated proteins 2 days after transfection (left). Arrows indicate vesicles. Scale bars, 10 μm. Insets show enlargements. Scale bars, 2 μm. Aggregate densities (right). (**D**) First frames and kymographs of PrP^PG14^-EGFP vesicle and aggregate transport in WT and *Kif5c^−/−^* axons. Scale bars, 10 mm. (**E**) Net velocities of PrP^PG14^-mCh vesicles and aggregates. (**F**) Densities, (**G**) population breakdown, (**H**) flux, and (**I**) percent time in motion/paused of PrP^PG14^-EGFP vesicles in WT and *Kif5c^−/−^* axons. Numbers of vesicles are inside the bars (H). Ant., anterograde; Ret., retrograde; Rev., reversals; Stat., stationary. (A, C, and E) Bars represent means ± SEM. Numbers of aggregates/tracks are inside the bars. **P < 0.01 and ***P < 0.001; ns, not significant; Student’s *t* test, Šidák correction. (F to I) Bars represent means ± SEM. *P < 0.05, **P < 0.01, and ***P < 0.001; Student’s *t* test. #, number

To characterize mutant PrP aggregate formation in axons, we established an in cellulo system in primary murine hippocampal neurons transiently expressing fluorescently tagged or untagged fusions of a PrP mutant harboring a nine octapeptide insertion called PrP^PG14^ (fig. S1A). This mutant was previously associated with dementia and ataxia in humans and with neurological illness in mouse disease models (*24*, *25*). Expression of PrP was under the Mouse PrP (MoPrP) promoter (*26*) and contained the endogenous PrP secretory signal sequence (SS) and GPI anchor sequence to ensure proper translocation to the ER and delivery to the plasma membrane, respectively. Live imaging showed prominent PrP^PG14^ axonal aggregate inclusions 1 day after the expression of PrP^PG14^-mCh (mCherry), PrP^PG14^-EGFP (enhanced green fluorescent protein), or untagged PrP^PG14^, and these increased over time compared to small vesicles observed in axons of PrP^WT^-mCh–expressing neurons (Fig. 1A and fig. S1B). These observations are consistent with previous studies showing intra-axonal aggregates in a PrP^PG14^-EGFP transgenic mouse model (*11*). Correlative fluorescence and scanning electron microscopy revealed localization of PrP^PG14^-mCh inclusions to axonal dystrophic swellings averaging 1.57 ± 0.13 μm in length and 0.54 ± 0.05 μm in width (means ± SEM) (Fig. 1B). PrP^PG14^-EGFP inclusions formed in differentiated Neuro2a (N2a) cells (fig. S1C), where PrP^PG14^-mCh exhibited limited resistance to proteinase K (PK) (fig. S1D), as shown previously (*27*), suggesting that this mutant partly and constitutively misfolded upon transient expression. Prominent intra-axonal inclusions were also generated in axons of neurons expressing PrP^D177N(M128)^-mCh (Fig. 1C), a point mutation (D178N/M129 in humans) that causes prion fatal familial insomnia (*28*), indicating that axonal aggregates are a generalized feature of at least some familial PrP mutations and not unique to PrP^PG14^.

**Fig. 2.**
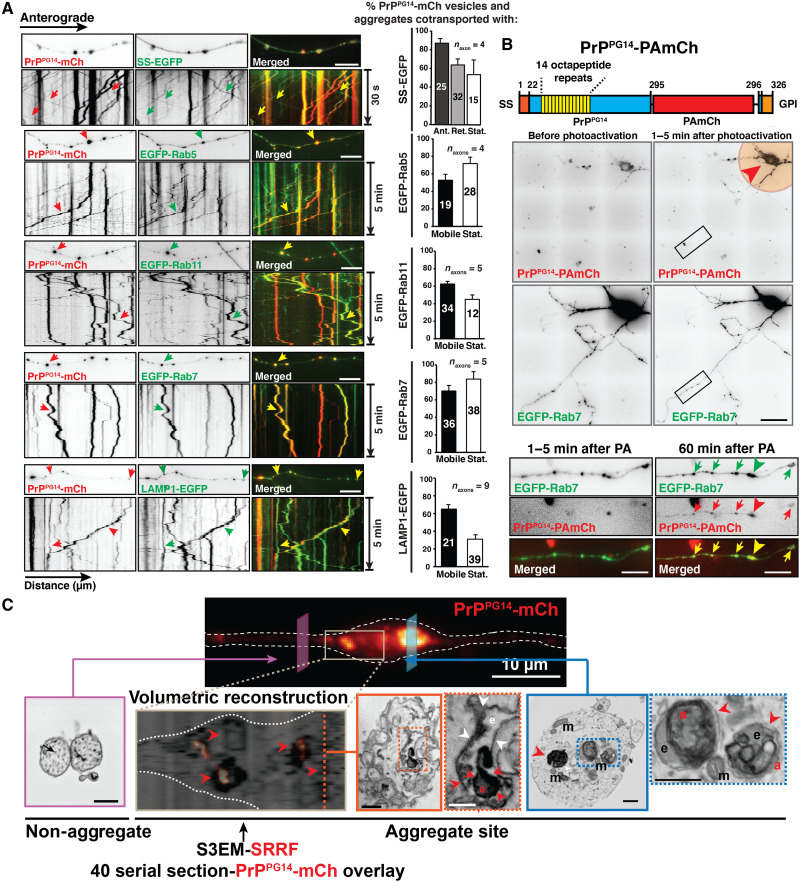
Golgi-derived PrP^PG14^ resides in axonal endolysosomes. (**A**) First-frame images and kymographs of time-lapse movies of axons coexpressing PrP^PG14^-mCh and various compartment markers (left): SS-EGFP, EGFP-Rab5, EGFP-Rab11, EGFP-Rab7, or LAMP1-EGFP. Arrows point to cotransport. Quantitation of cotransport (right). Bars represent means ± SEM. Vesicle or aggregate numbers are shown inside the bars. Scale bars, 10 μm. (**B**) Schematic of PrP^PG14^-PAmCh construct (top). GPI, GPI anchor. Representative images of a hippocampal neuron cotransfected with PrP^PG14^-PAmCh and EGFP-Rab7 before and after photoactivation (pink circle, arrowhead) of the cell body with a 405-nm laser (middle). Scale bar, 50 μm. Enlargements of inset in middle panels (bottom). Scale bar, 10 μm. Arrows indicate colocalization. Arrowheads points to endoggresomes. Number of neurons = 3. (**C**) Correlative fluorescence (top) and S3EM representative cross sections (bottom). Volumetric 3D reconstruction through the axonal PrP^PG14^-mCh aggregate site is shown with superresolution radial fluctuation (SRRF) fluorescence overlay. Red arrowheads point to PrP^PG14^-mCh-positive aggregates (a). White arrowheads point to endosomes (e). m, mitochondria; nonagg, nonaggregate. Scale bar (top panel of correlative image), 10 mm. Other scale bars, 500 nm.

In addition to large aggregates, high-resolution live imaging revealed the presence of small PrP^PG14^-EGFP vesicles that underwent active bidirectional transport in axons (Fig. 1D and movie S1). Single-particle image analysis (*29*) revealed that, 2 days after expression, most motile PrP^PG14^-mCh or PrP^PG14^-EGFP vesicles moved in the anterograde direction toward the synapse, as observed for PrP^WT^-mCh vesicles (Fig. 1, D and E; fig. S2, A and B; and movie S2) [transport parameters are defined in (*29*)] and as previously characterized for yellow fluorescent protein (YFP)–PrP^C^ vesicles (*30*). In contrast, PrP^PG14^-mCh aggregates remained largely stationary or moved with net velocities that were 20 to 50 times slower (0.05 to 0.1 μm/s) than those of vesicles (0.5 to 4 μm/s) (vesicles versus aggregates are defined in Materials and Methods; Fig. 1, D and E, and fig. S2B). Depletion of kinesin-1C (KIF5C), an anterograde motor that we previously identified as required for the movement of YFP-PrP^C^ vesicles in axons (*30*), resulted in an overall impairment of anterograde movement of PrP^PG14^-mCh or PrP^PG14^-EGFP vesicles toward the axonal termini. Defects included significant reductions in the densities and flux (number of vesicles per time) of anterograde-moving vesicles and an increased number of stalled vesicles (Fig. 1, F to H). Vesicles that continued to move spent less time moving toward the synapse, moved slower, and paused for longer periods during an anterograde run (Fig. 2I and fig. S2, B to D). These observations indicate that misfolded PrP^PG14^ is actively transported in vesicles by kinesin-1 in axons, where it also forms largely immobile aggregates at dystrophic swelling sites.

### Vesicular and aggregated mutant PrP reside in endolysosomes in axons

To identify the axonal membrane compartments containing misfolded PrP^PG14^, we imaged axons expressing PrP^PG14^-mCh and various endolysosomal markers. Live imaging and quantitative analysis revealed substantial colocalization and cotransport of PrP^PG14^-mCh vesicles and aggregates with EGFP-labeled Rab5, Rab11, Rab7, and LAMP1, markers for early endosomes, recycling endosomes, LEs, and endolysosomes, respectively (Fig. 2A and fig. S3A). Extensive colocalization was confirmed for PrP^PG14^-mCh vesicles and aggregates in axons fixed and stained with antibodies recognizing endogenous endosomal proteins including early endosomes (EEA1), LEs (Rab7), and endolysosomes [LAMP1 (lysosomal associated membrane protein 1)] (fig. S3B). Time-lapse live imaging also revealed extensive cotransport of PrP^PG14^-mCh vesicles with a fluorescently labeled fusion carrying only the SS of neuropeptide Y (NPY; referred to as SS-EGFP) (Fig. 2A), shown previously to exit the Golgi and traverse through the secretory pathway before cellular secretion (*31*), indicating that PrP^PG14^ particles are Golgi-derived. To directly test that Golgi-derived PrP^PG14^ vesicles entered to the axon in endolysosomes from the soma, we performed live imaging of a photoactivatable (PA) PrP^PG14^-PAmCh fusion following irradiation of the soma with a 405-nm laser in neurons also expressing EGFP-Rab7 (Fig. 2B). We observed the rapid transition and cotransport of PrP^PG14^-PAmCh vesicles with EGFP-Rab7–positive LEs from the soma into the axon, where they also colocalized with large PrP^PG14^-PAmCh aggregates after 1 hour (Fig. 2B). These data demonstrate the direct soma-to-axon entry of PrP^PG14^-PAmCh vesicles and suggest that PrP^PG14^/Rab7 (LE)–positive vesicles contribute to the buildup of large aggregates in axons.

We next sought to determine whether misfolded PrP^PG14^ aggregates resided in endosomes. We first tested whether PrP^PG14^ cofractionated biochemically with Rab7 from detergent-free sucrose-floated membrane fractions (fig. S3C). We observed PrP in floated vesicle fractions of N2a cells transfected with either PrP^WT^-mCh or PrP^PG14^-mCh using antibodies against PrP or mCh (fig. S2, C and D). Floated fractions were positive for Rab7, confirming the biochemical isolation of LEs (fig. S2, C and D). To ascertain that floated membrane fractions contained misfolded and aggregated PrP^PG14^, we subjected these fractions to PK digestion and to an insolubility assay using nondenaturing detergents, two assays used to test for misfolded PrP conformations, including of PrP^PG14^ (*18*, *25*). Western blots showed that PrP^PG14^-mCh isolated in floated membrane fractions was partially resistant to PK digestion and more detergent insoluble compared to PrP^WT^-mCh (fig. S2, E and F), indicating that vesicular PrP^PG14^ in primary neurons acquires major biochemical properties characteristic of conformationally misfolded and aggregated PrP (*11*, *27*).

To further determine whether PrP^PG14^ aggregates localized within endosomal membrane compartments, we performed correlative light and single or volumetric three-dimensional (3D) reconstructions using serial sectioning scanning electron microscopy (S3EM) of aggregate versus nonaggregate regions. This approach allows the precise mapping of PrP^PG14^-mCh signal to structures in the S3EM images. Compared to nonaggregate regions, cross sections that mapped to PrP^PG14^-mCh–positive aggregate sites revealed large electron-dense structures that resided within membrane compartments resembling endosomes, measuring an average length of 1286.9 ± 195.7 nm (mean ± SEM; *n* = 7) (Fig. 2C). Collectively, these data indicate that PrP^PG14^ is actively transported into the axon in Golgi-derived endolysosomal compartments that contribute to the formation of aggregates inside endomembrane structures that we term endoggresomes. The presence of endoggresomes suggests mechanisms that actively promote mutant PrP aggregate formation in axons and/or of those that impair their clearance.

### Transient access of misfolded PrP^PG14^ to the axonal cell surface contributes to endoggresome formation

**Fig. 3.**
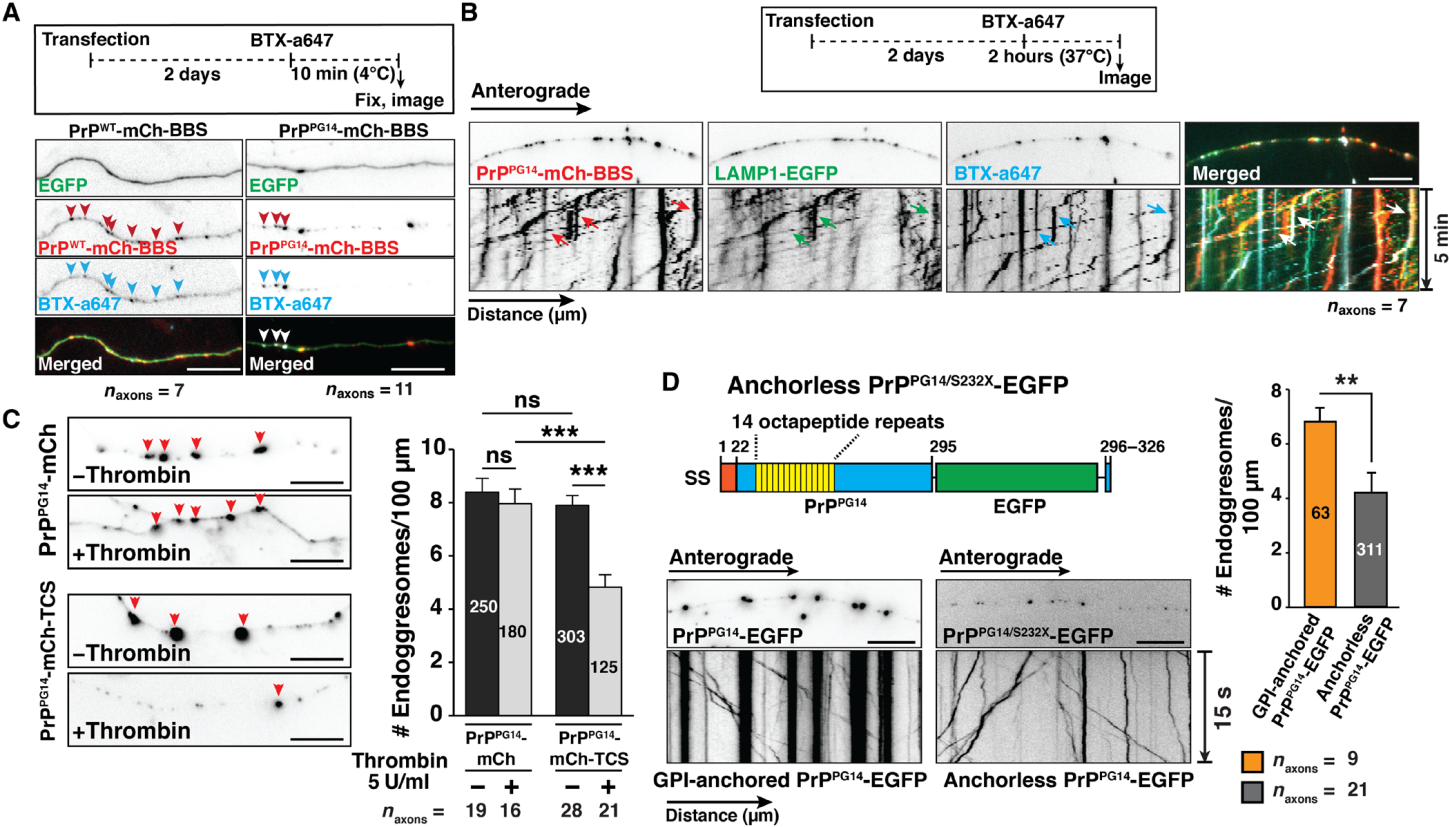
Endoggresome formation requires the rapid transit of mutant PrP to the axonal cell surface. (**A**) Outline of 10-min BBS internalization assay (top). Representative images of axons expressing EGFP and PrP^WT^-mCh-BBS or PrP^PG14^-mCh-BBS and labeled with BTX-a647 (bottom). Arrowheads point to colocalization. Scale bars, 10 μm. (**B**) Outline of 2-hour BBS internalization assay (top). Representative first frames of time-lapse movies and kymographs of axons expressing PrP^PG14^-mCh-BBS and LAMP1-EGFP, and labeled with BTX-a647. Arrows point to cotransport. Scale bar, 10 μm. (**C**) Representative images of axons expressing PrP^PG14^-mCh or PrP^PG14^-mCh-TCS, with or without thrombin treatment (left). Arrowheads point to endoggresomes. Scale bars, 10 μm. Quantitation of endoggresome densities (right). Numbers of endoggresomes are shown inside the bars. Bars represent means ± SEM. ***P < 0.001; Student’s *t* test, Tukey’s correction. (**D**) Schematic diagram of anchorless PrP^PG14/S232X^-EGFP construct (top left). Representative images and kymographs of hippocampal axons expressing PrP^PG14^-EGFP or PrP^PG14/S232X^-EGFP (bottom left). Scale bars, 10 μm. Quantitation of endoggresome densities (right). Numbers of endoggresomes are shown inside the bars. Bars represent means ± SEM. **P < 0.01; Student’s *t* test.

PrP is a secreted protein that transits to the cell surface (*20*). To determine whether PrP^PG14^ expressed in cultured neurons traveled along axons and accessed the plasma membrane along the axon en route to aggregation, we transfected neurons with PrP^PG14^-mCh or PrP^WT^-mCh fusion constructs containing a bungarotoxin binding sequence (BBS; referred to as PrP^PG14^-mCh-BBS or PrP^WT^-mCh-BBS; fig. S4A). The media of these neurons was treated with Alexa Fluor 647-labeled bungarotoxin (BTX) [BTX-a647 (*32*)] for 10 min at 4°C to inhibit active endocytosis, and neurons were fixed without permeabilization before imaging (fig. S4B). BTX-a647 signal was colocalized with a subset of PrP^PG14^-mCh-BBS-positive puncta along axons (Fig. 3A), indicating that the latter accessed the axonal plasma membrane. BTX-a647 labeling was specific, as a647 signal was not observed in neurons expressing PrP^WT^-mCh or PrP^PG14^-mCh without the BBS sequence (fig. S4C). To determine whether PrP^PG14^ was sorted into LEs following internalization, neurons coexpressing PrP^PG14^-mCh-BBS and LAMP1-EGFP were treated with BTX-a647 for 2 hours at 37°C before live imaging or fixation and permeabilization (fig. S4B). We observed extensive colocalization and cotransport between intra-axonal PrP^PG14^-mCh-BBS, LAMP1-EGFP, and BTX-a647 signal (Fig. 3B), indicating that PrP^PG14^-mCh-BBS was sorted into LEs after endocytosis. BTX-a647 punctate signal was observed along the length of axons, revealing that PrP^PG14^ undergoes dynamic bouts of exocytosis and endocytosis throughout the axonal surface. The addition of the BBS sequence did not alter endoggresome densities in axons (fig. S4D). Inhibition of endocytosis with dynasore, a dynamin GTPase inhibitor, resulted in a significant, albeit partial decrease in internalization of BTX-a647-labeled PrP^PG14^-mCh particles (fig. S4E), suggesting that PrP^PG14^-mCh is partly internalized by clathrin-dependent pathways.

To determine whether targeting of PrP^PG14^-mCh to the cell surface was required to form intra-axonal aggregates, we quantified endoggresome densities in axons of neurons expressing PrP^PG14^-mCh tagged or not with a thrombin cleavage sequence (TCS; referred as PrP^PG14^-mCh-TCS) and treated or not with thrombin protease (5 U/ml) for 48 hours (fig. S4F). Thrombin treatment resulted in significantly reduced densities of PrP^PG14^-mCh-TCS endoggresomes compared to untagged PrP^PG14^-mCh (Fig. 3C), indicating that access to the plasma membrane contributes substantially to the formation of endoggresomes in axons. We further tested this cell surface requirement by expressing an anchorless PrP^PG14^ double mutant (PrP^PG14/S232X^-EGFP) that is unable to associate with membranes (*21*). While small PrP^PG14/S232X^-EGFP vesicles were actively transported in axons, the density of endoggresomes was largely and significantly reduced (Fig. 3D), suggesting that, upon secretion, PrP^PG14/S232X^-EGFP is released in the absence of the GPI anchor and thus cannot endocytose and contribute to aggregation. These data show that the transient transit of PrP^PG14^ to the cell surface and subsequent rapid endocytosis all along the length of axons contribute to the sorting of Golgi-derived PrP^PG14^ into endosomes to form endoggresomes in LEs.

### An Arl8b/kinesin-1/HOPS pathway drives PrP^PG14^ endoggresome formation in axons

**Fig. 4.**
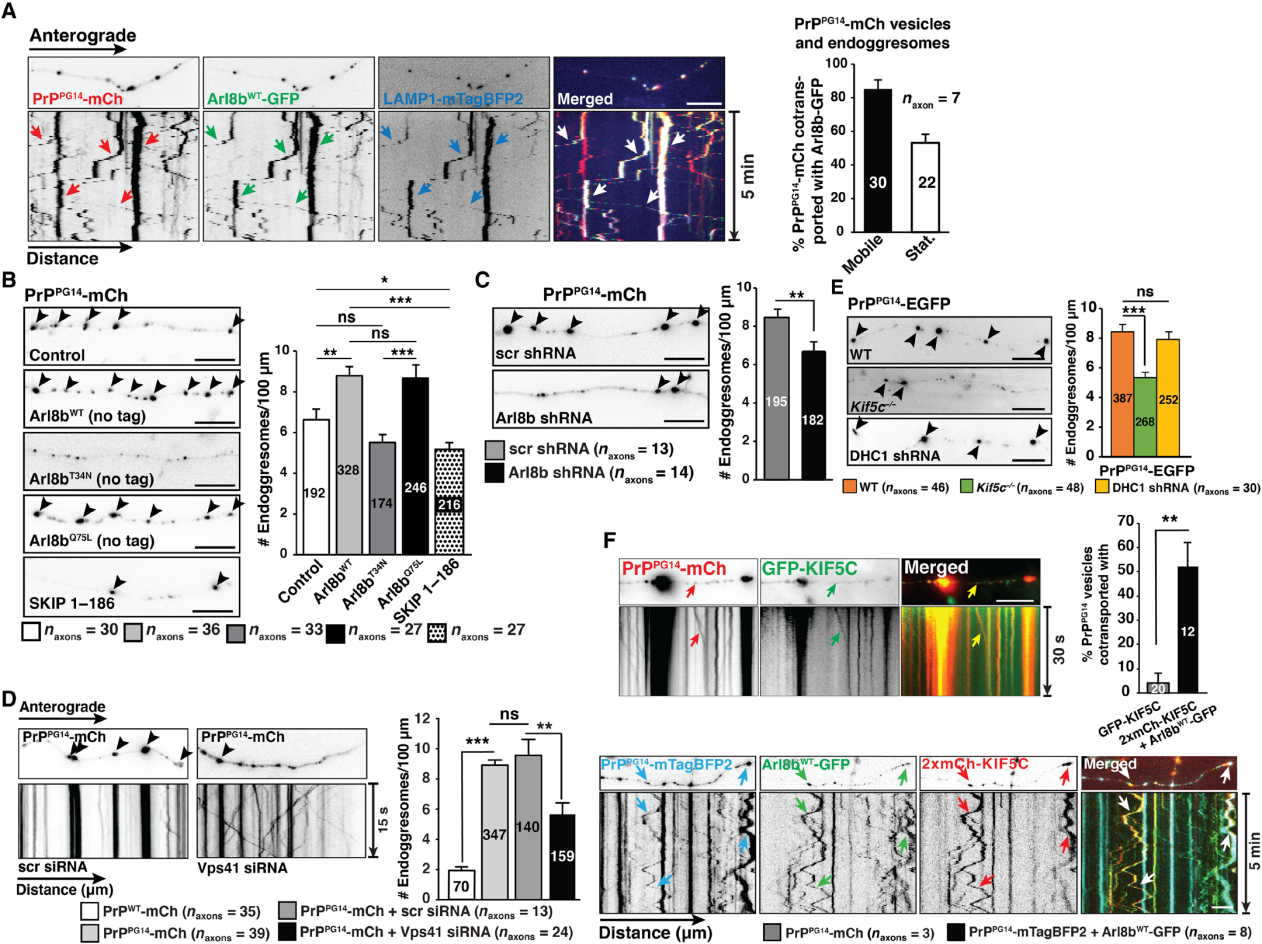
The axonal rapid endosomal sorting and transport-dependent aggregation pathway drives mutant PrP endoggresome formation. (**A**) First frames of time-lapse movies and kymographs of axons expressing the indicated markers (left). Arrows indicate cotransport. PrP^PG14^-mCh and Arl8b^WT^-GFP percentage contransport (right). Vesicle/endoggresome numbers are inside the bars. Bars represent means ± SEM. (**B**) Images of PrP^PG14^-mCh axons coexpressing the indicated markers. Control, empty vector. Arrowheads indicate endoggresomes. Endoggresome densities (right). Endoggresome numbers are inside the bars. (**C**) Images of axons from neurons expressing PrP^PG14^-mCh treated with scrambled (scr) or Arl8b shRNAs (left). Arrowheads indicate endoggresomes. Endoggresome densities (right). Endoggresome numbers are inside the bars. (**D**) First frames of time-lapse movies and kymographs of axons coexpressing PrP^PG14^-mCh and scr or Vps41 siRNAs (left). Arrowheads indicate endoggresomes. Endoggresome densities (right). Endoggresome numbers are inside the bars. (**E**) Images of PrP^PG14^-EGFP endoggresomes in WT, *Kif5c^−/−^*, and DHC1 shRNA axons (left). Arrowheads indicate endoggresomes. Endoggresome densities (right). Endoggresome numbers are inside the bars. (**F**) First frames of time-lapse movies and kymographs of axons expressing PrP^PG14^-mCh and GFP-KIF5C (top left), as well as PrP^PG14^-mTagBFP2, Arl8b^WT^-GFP, and 2xmCh-KIF5C (bottom). Arrows indicate cotransport. Percentage of cotransport (top right). Vesicle numbers are inside the bars. (B to D) Bars represent means ± SEM. *P < 0.05, **P < 0.01, and ***P < 0.001; Student’s *t* test, Šidák correction. (E and F) Bars represent means ± SEM. ***P < 0.001; **P < 0.01; Student’s *t* test. Scale bars, 10 μm.

Two observations led us to hypothesize that direct Golgi-to-LE and/or LE homotypic fusion (*33*–*35*) also contributed to endoggresome biogenesis in axons. First, axonal PrP^PG14^ particles were endocytosed from the cell surface into single endosomes (Fig. 3B), but, over a period of 2 to 5 days, PrP^PG14^ was observed in enlarged LEs (Figs. 1, A and B, and 2C), suggesting fusion of endosomes after endocytosis. Second, preventing PrP^PG14^ internalization along axons reduced but did not abolish the number of PrP^PG14^ endoggresomes (Fig. 3C), suggesting the presence of parallel endoggresome-forming pathways. As Arl8b orchestrates endolysosomal trafficking and fusion (*6*), we first evaluated whether it associated with PrP^PG14^ endosomal vesicles to direct their homotypic fusion in axons toward endoggresome formation. Live imaging showed that most PrP^PG14^-mCh vesicles co-migrated with Arl8b^WT^-GFP (80%) or with active GTP-bound Arl8b^Q75L^-GFP and LAMP1-mTagBFP2 particles, but not with soluble dominant-negative guanosine diphosphate (GDP)–bound Arl8b^T34N^-GFP (Fig. 4A and fig. S5A). Two days after co-expression, we observed Arl8b^WT^-mCh and PrP^PG14^-mTagBFP2 vesicle colocalization in the soma, and these vesicles co-transported with SS (from NPY)–EGFP into the axon (fig. S5, B and C), suggesting that Arl8b loaded onto post-Golgi PrP^PG14^ vesicles and entered the axon as a complex. Endogenous Arl8b, as recognized with an anti-Arl8b antibody, also colocalized with PrP^PG14^ vesicles in axons (fig. S9C). To evaluate the role of Arl8b on endoggresome formation, we expressed inactive untagged GDP-bound Arl8b^T34N^, or Arl8b function was reduced using previously validated short hairpin RNAs (shRNAs) (fig. S5D) (*36*). Endoggresome densities were significantly decreased in axons of neurons treated with Arl8b shRNAs and in those expressing GDP-bound inactive Arl8b^T34N^, while overexpression of untagged Arl8b^WT^ or GTP-active Arl8b^Q75L^ increased endoggresome densities in axons (Fig. 4, B and C).

We next evaluated the mechanistic basis of Arl8b-mediated endoggresome formation. We first tested whether Arl8b recruited its effectors SifA and kinesin-interacting protein (SKIP), KIF5C, and Vps41 to PrP^PG14^ vesicles (*7*). Live imaging showed extensive cotransport between vesicles carrying PrP^PG14^-mTagBFP2, Arl8b^WT^-mCh, and EGFP-hSKIP; PrP^PG14^-mCh and EGFP-hSKIP; Arl8b^WT^-mCh and EGFP-hSKIP; and PrP^PG14^-mCh and EGFP-Vps41 (fig. S5E). To next determine the role of Arl8b effectors in the trafficking, fusion, and aggregation of PrP^PG14^ in axons, we reduced the function of Vps41 using small interfering RNAs (siRNAs) (fig. S5, F and G), and the function of SKIP was inhibited using a peptide comprising amino acids 1 to 186 (SKIP 1–186) previously shown to be required to bridge interactions between Arl8b and kinesin-1 (*37*). We observed significantly decreased endoggresome densities following reduction and inhibition of Vps41 and SKIP (Fig. 4, B and D). Furthermore, reducing Vps41 resulted in small and less stationary PrP^PG14^-mCh vesicles in the soma and axons (Fig. 4D and fig. S5, G and H), suggesting decreased vesicle fusion, and showing restored vesicular motility. To determine the role of kinesin-1 in endoggresome formation, we quantitated endoggresome densities in axons of kinesin-1C knockout (KO) (*Kif5c^−/−^*) mice, kinesin light chain 1 (KLC1) KO (*Klc1^−/−^*) mice, and conditional kinesin-1B KO (*Kif5b^pflox/pflox^*) mice (*38*). The latter two kinesin-1 subunits were previously implicated in the anterograde transport of YFP-PrP^C^ vesicles (*30*). Endoggresome densities were significantly decreased 2 days after PrP^PG14^-mCh transfection, in axons from *Kif5c*^−/−^, *Klc1*^−/−^, and *Kif5b^pflox/pflox^* neurons—the latter in a cre-adenovirus multiplicity of infection (MOI) dose-dependent manner—compared to WT hippocampal axons (Fig. 4E and fig. S6, A to C). Decreased endoggresome densities were sustained for at least 10 days in *Kif5c*^−/−^ axons (fig. S6D). Overexpression of EGFP-KIF5C in *Kif5c^−/−^* neurons transfected with PrP^PG14^-mCh restored higher axonal endoggresome densities, demonstrating that decreased densities were due to the specific removal of KIF5C (fig. S6E). Notably, aggregate densities in axons of *Kif5c^−/−^* neurons expressing PrP^D177N(M128)^-mCh were also significantly lower (fig. S6F), indicating a generalized requirement of kinesin-1 for generating intra-axonal endoggresomes of various mutant PrPs. To test whether endoggresome formation in axons was the result of generalized transport impairments, the function of the main neuronal retrograde motor cytoplasmic dynein heavy chain 1 (DHC1) was reduced using previously validated shRNAs (*30*). Despite impairing PrP^PG14^-mCh vesicle transport, and thus identifying DHC1 as a retrograde motor for these vesicles (fig. S6G), reducing DHC1 did not alter PrP^PG14^-EGFP endoggresome densities (Fig. 4E).

To determine whether Arl8b drives endoggresome formation via recruitment of kinesin-1 to PrP^PG14^ vesicles, we performed live imaging of vesicle transport of neurons coexpressing PrP^PG14^-mTagBFP2 and 2xmCh-KIF5C in the presence or absence of Arl8b^WT^ overexpression. Overexpression of Arl8b^WT^-GFP significantly increased the percentage of PrP^PG14^-mTagBFP2 vesicles that cotransported with 2xmCh-KIF5C (Fig. 4F), suggesting recruitment of 2xmCh-KIF5C by Arl8b. Moreover, flux and densities of PrP^PG14^-mCh vesicles were significantly enhanced in axons upon Arl8b^WT^ overexpression (fig. S6H). Recruitment of KIF5C by Arl8b depended on Arl8b GTPase activity (fig. S6I). Together, these data indicate that GTP-active Arl8b^WT^ loads onto Golgi-derived PrP^PG14^ vesicles, recruits SKIP, kinesin-1, and Vps41, and acts as a key driver of the anterograde movement and fusion of PrP^PG14^ vesicles in the axon, promoting endoggresome formation. Thus, we term this process Axonal Rapid Endosomal Sorting and Transport-dependent Aggregation (ARESTA).

### Direct and indirect ARESTA converge on Arl8b to form axonal endoggresomes

As endoggresome formation occurs by first accessing the axonal cell surface (Fig. 3) or by direct transport and fusion (Fig. 4), we investigated whether these pathways act independently or in concert via Arl8b, by overexpressing Arl8b^WT^ to promote endoggresome formation while inhibiting the uptake of cell surface PrP^PG14^. Live imaging of neurons expressing PrP^PG14^-mCh-TCS and simultaneously transfected with Arl8b^WT^-GFP revealed the same increased endoggresome densities in axons treated or not with thrombin (fig. S6J). These observations revealed that Arl8b^WT^ overexpression alone was sufficient to promote endoggresome formation, bypassing the contribution of endocytosed PrP^PG14^, and suggest that aggregation via direct fusion and via internalization act in parallel.

We next evaluated whether Arl8b associated with PrP^PG14^ vesicles endocytosed from the cell surface to drive endoggresome formation. Live imaging of axons expressing PrP^PG14^-mCh-BBS and Arl8b^WT^-GFP and treated in the media with BTX-a647 resulted in extensive cotransport between these three vesicle populations (fig. S6K). Thus, Arl8b loads onto PrP^PG14^ vesicles that are endocytosed from the cell surface along the axon, as well as to post-Golgi vesicles (fig. S5, A and E), to mediate PrP^PG14^ endosome fusion. These “indirect” and “direct” ARESTA pathways act in parallel and converge on Arl8b, a key regulator of mutant PrP endoggresome formation.

### Misfolded PrP is degraded in the soma but persists in axons because of impaired retrograde transport and diminished lysosomal degradation capacity

**Fig. 5.**
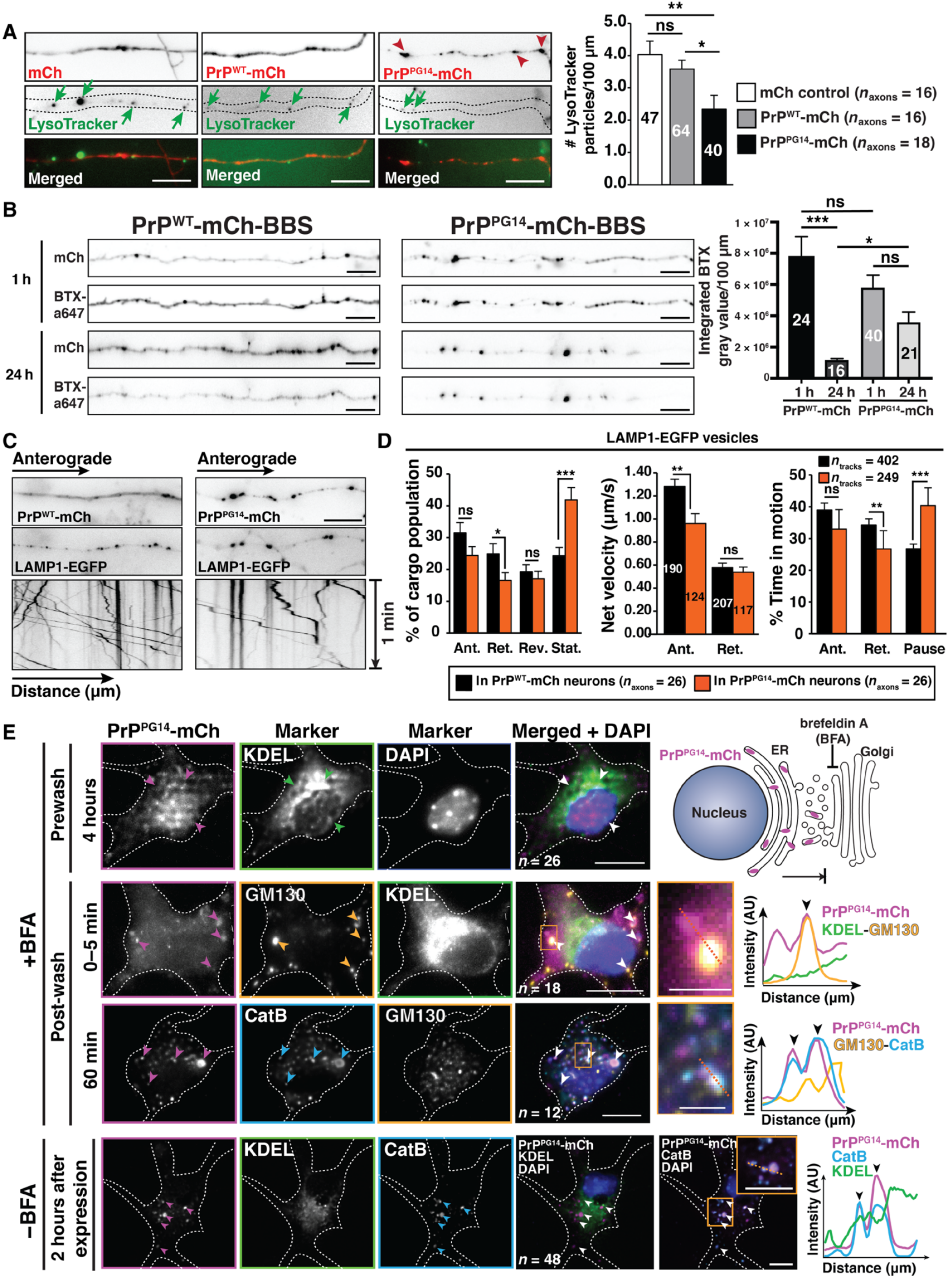
Endoggresomes are maintained in axons because of impaired retrograde transport and lysosomal degradation. (**A**) Images of LysoTracker-positive vesicles in mCh, PrP^WT^-mCh, or PrP^PG14^-mCh-expressing axons (left). Arrows indicate vesicles, and arrowheads indicate endoggresomes. Scale bars, 10 μm. LysoTracker vesicle densities (right). Vesicle numbers are inside the bars. (**B**) Images of BTX-a647–labeled axons 1 and 24 hours after labeling (left). Scale bars, 10 μm. BTX signal intensity (right). Axon numbers are inside the bars. PrP^WT^-mCh-BBS: four and three replicates for 1 and 24 hours, respectively; and PrP^PG14^-mCh-BBS: three and two replicates for 1 and 24 hours, respectively. (**C**) Images of PrP^WT^-mCh or PrP^PG14^-mCh axons (top), and LAMP1-EGFP first frames of a time-lapse movie (middle) and kymographs (bottom). Scale bar, 10 μm. (**D**) LAMP1-EGFP vesicles: Population percentage (left), net velocities (middle), and percent time in motion/paused (right). Track numbers are inside the bars. (**E**) Images of soma of neurons expressing PrP^PG14^-mCh and stained with antibodies against the indicated markers. Arrowheads indicate colocalization. Schematic of the BFA assay (top right). Line scans are intensity profiles of regions indicated by dotted lines inside insets. Scale bars (main panels), 10 μm. Scale bars (insets), 250 nm. (A, B, and D) Bars represent means ± SEM. *P < 0.05, **P < 0.01, and ***P < 0.001; Student’s *t* test, Tukey’s correction. DAPI, 4′,6-diamidino-2-phenylindole; AU, arbitrary units.

The axon has limited local degradation capacity, with an increasing gradient of acidification of endosomal organelles from distal to proximal axonal regions (*39*, *40*). Thus, most cargos require active retrograde transport for degradation upon fusion with lysosomes enriched in the soma (*5*, *41*, *42*). To determine how PrP^PG14^ endoggresomes persist in axons, we tested for local axonal degradation impairments and/or failure of PrP^PG14^ vesicles to undergo retrograde transport. Several observations suggested local axonal deficits in lysosomal maturation and clearance. First, axonal endoggresomes increased over time compared to PrP^WT^-mCh–positive puncta (Fig. 1A), suggesting impaired in situ degradation. Second, treatment of PrP^PG14^-mCh neurons with bafilomycin A1 (BafA1), an inhibitor of a lysosomal V–adenosine triphosphatase (ATPase) proton pump that acidifies vesicles (*43*), did not increase endoggresome densities compared to dimethyl sulfoxide (DMSO)–treated controls (fig. S7A), suggesting the absence of actively degradative endolysosomal compartments. As a positive control, PrP^WT^-mCh neurons treated with BafA1 showed a significant increase in endoggresome densities compared to DMSO controls, indicating inhibition of acidification (fig. S7A). Consistent with these observations, LysoTracker signal was not readily observed in PrP^PG14^-mCh axons (Fig. 5A and fig. S7B). Third, BTX-a647 treatment and wash-off of neurons expressing PrP^WT^-mCh-BBS resulted in reduced BTX-a647 axonal fluorescence intensity after 24 hours, compared to the steady BTX-a647 axonal fluorescence levels in neurons expressing PrP^PG14^-mCh-BBS (Fig. 5B and fig. S7C), suggesting that a day after treatment, endocytosed PrP^PG14^-mCh-BBS molecules were not significantly degraded in lysosomes compared to PrP^WT^-mCh-BBS–internalized molecules.

To determine whether retrograde PrP^PG14^ vesicle transport was impaired, live imaging and quantitative analysis showed that, in addition to the largely stationary or slow-moving PrP^PG14^-mCh larger endoggresomes (Fig. 1E and fig. S2B), PrP^PG14^-EGFP vesicles also moved slower and with less processivity in anterograde and retrograde directions compared to PrP^WT^-EGFP vesicles (fig. S7D). To further test whether overall retrograde transport was impaired in PrP^PG14^-mCh neurons, LAMP1-EGFP vesicle transport was imaged live. In PrP^PG14^-mCh axons, the proportion of stationary LAMP1-EGFP vesicles was significantly increased and fewer vesicles moved in the retrograde direction (Fig. 5, C and D). LAMP1-EGFP vesicles also spent more time paused and less time moving toward the soma in PrP^PG14^-mCh versus PrP^WT^-mCh axons (Fig. 5D). Thus, PrP^PG14^ vesicles and endoggresomes, as well as LAMP1-positive LEs, are not properly transported toward the soma, nor are they actively degraded in axonal lysosomes, contributing to their sustained presence in axons.

As lysosomes are enriched in the soma (*5*), we evaluated whether PrP^PG14^-mCh was actively degraded in the soma by systematically analyzing the trafficking itinerary of misfolded PrP^PG14^ immediately upon neuronal expression. Live imaging of neurons expressing PrP^PG14^-mCh and treated with brefeldin A (BFA), an inhibitor of the secretory pathway (*44*), showed ER retention of PrP^PG14^-mCh for at least 6 hours (Fig. 5E and fig. S8A). Untreated or post–BFA wash neurons showed rapid, efficient, and successive translocation within minutes, and by 2 hours of expression, of PrP^PG14^-mCh from the ER to the Golgi and to compartments positive for cathepsin B (CatB), an acidic hydrolase required for lysosomal substrate degradation (Fig. 5E and fig. S8A). Neurons expressing PrP^PG14^-mCh-BBS and treated with BTX-a647 in the media showed colocalization of these vesicles with LAMP1-EGFP (fig. S8B), indicating internalization into LEs. This labeling was specific, as neurons transfected with PrP^PG14^ fusion constructs without the BBS tag did not reveal any BTX-a647 signal (fig. S8C). Furthermore, PrP^PG14^-mTagBFP2 vesicles colocalized with LysoTracker and Magic Red in the soma (fig. S8D), a fluorogenic substrate that activates upon cleavage by active CatB (*45*). Live imaging showed the active disappearance of PrP^PG14^-mCh puncta that colocalized with LAMP1-EGFP, suggesting active degradation in the soma via the cell surface (fig. S8E and movie S3). To further test whether PrP^PG14^ underwent lysosomal degradation in the soma, we treated neurons expressing PrP^WT^-mCh or PrP^PG14^-mCh and exposed to LysoTracker Green with either BafA1 or DMSO. Quantitative imaging showed a significant increase in fluorescence intensity in the soma of PrP^WT^-mCh– and PrP^PG14^-mCh–expressing neurons treated with BafA1, compared to those treated with DMSO (fig. S8F), indicating that PrP undergoes somatic lysosomal-based degradation. Because this clearance pathway resembles a previously characterized rapid ER stress–induced export (RESET) pathway in non-neuronal cells (*21*), we evaluated whether PrP^PG14^ expression turned on ER stress in neurons. Coexpression of PrP^PG14^-EGFP and an ER stress transcriptional reporter (ERSE-mCh) (*46*) resulted in significantly increased signal compared to that observed in neurons expressing either soluble EGFP or PrP^WT^-EGFP (fig. S8G), indicating constitutive ER stress activation. Thus, while secreted misfolded PrP is actively cleared in somatic lysosomes by a process similar to RESET, PrP^PG14^ vesicles and endoggresomes are neither effectively transported toward the soma nor actively degraded in the axon, contributing to their sustained axonal presence.

### Arl8b is a key determinant of PrP^PG14^ axonal entry

Because Arl8b is an endolysosomal GTPase, to determine whether Arl8b tags PrP^PG14^ endolysosomes destined for axonal entry versus PrP^PG14^ vesicles shuttled to lysosomes in the soma for degradation, we followed the ER-to-LE itinerary of PrP^PG14^-mTagBFP2 (or PrP^PG14^-mCh) and Arl8b in the soma and proximal axons of neurons treated or not with BFA. Live imaging showed that, while PrP^PG14^-mTagBFP2 (or PrP^PG14^-mCh) translocated from the ER to the Golgi and to LAMP1-mTagBFP2–positive endolysosomes immediately after BFA wash, as observed earlier (Fig. 5E), colocalization between PrP^PG14^-mTagBFP2 (or PrP^PG14^-mCh) and Arl8b puncta was not observed during the ER-to-LE itinerary, but only 2 hours after transfection (fig. S9A). This time period was after PrP^PG14^ was observed to transit to lysosomes (Fig. 5E). To determine whether Arl8b associated with PrP^PG14^ vesicles as they internalized to LEs from the somatic cell surface, neurons were cotransfected with PrP^PG14^-mCh-BBS and LAMP1-mTagBFP2, treated in the medium with BTX-a647, and fixed and stained with an antibody against endogenous Arl8b. Imaging of the soma revealed the colocalization of PrP^PG14^-mCh-BBS and LAMP1-mTagBFP2 vesicles that were also positive for BTX-a647 signal but that were negative for Arl8b (fig. S9B). Instead, colocalization of Arl8b with PrP^PG14^-mCh-BBS, BTX-a647 signal, and LAMP1-mTagBFP2 vesicles occurred in the proximal axon (fig. S9, B and C), consistent with our earlier observations in more distal axonal regions (fig. S6K), indicating that Arl8b does not recognize PrP^PG14^ vesicles that are internalized from the somatic cell surface. Thus, Arl8 is absent from endocytosed PrP^PG14^ vesicles undergoing lysosomal-based degradation in the soma, but uniquely loads onto post-Golgi PrP^PG14^ vesicles that enter the axon, suggesting that Arl8b is a key regulator of PrP^PG14^ axonal entry.

### PrP^PG14^ neurons display ARESTA-dependent impaired calcium intake dynamics and decreased neuronal viability

**Fig. 6.**
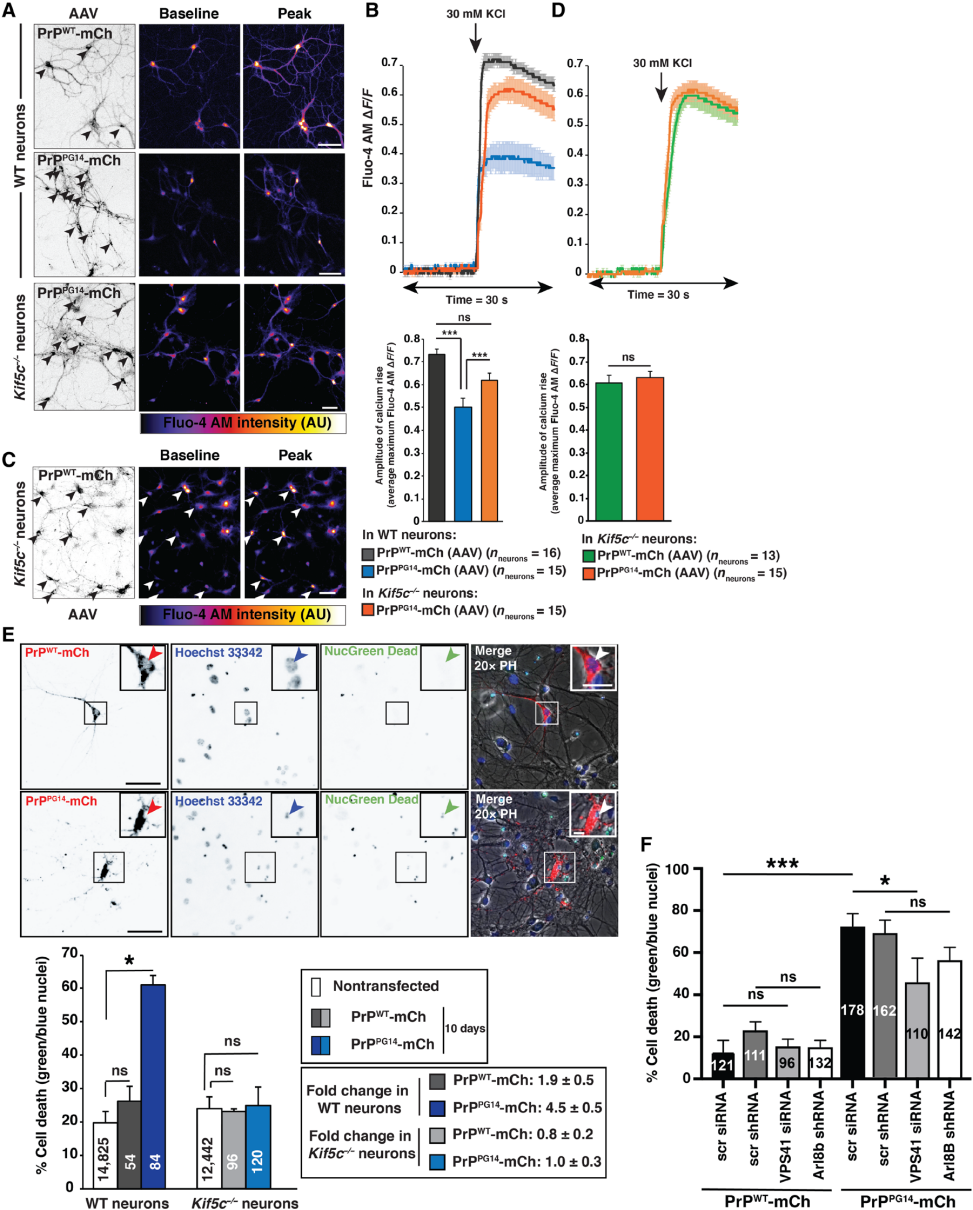
ARESTA-dependent impaired calcium intake dynamics and decreased neuronal viability of PrP^PG14^ neurons. (**A** and **C**) Images of PrP^WT^-mCh or PrP^PG14^-mCh AAV-transduced WT or *Kif5c*^−/−^ neurons (left). Arrowheads indicate the soma. Baseline and peak Fluo-4 AM intensities before (Baseline; middle) and after KCl treatment (Peak; right). Scale bars, 50 μm. (**B** and **D**) KCl-induced depolarization response curve from normalized Fluo-4 AM intensities from (A) and (C) (top). Arrows indicate the KCl treatment time. Average maximum amplitude of calcium rise (bottom). Bars represent means ± SEM. ***P < 0.001; Student’s *t* test, Tukey’s correction. (**E**) Images of neurons expressing PrP^WT^-mCh or PrP^PG14^-mCh and stained with Hoechst 33342 and NucGreen Dead (top). Arrowheads indicate nuclei. Insets are enlargements. Scale bars (main figures), 100 μm. Scale bars (insets), 20 μm. Percentage of cell death (bottom left). Cell numbers are inside the bars. Fold changes between indicated conditions (bottom right). Bars represent means ± SEM. *P < 0.05; Kruskal-Wallis test. PH, phase contrast. (**F**) Percentage cell death of neurons transduced with AAV8-PrP^WT^-mCh or AAV8-PrP^PG14^-mCh and treated with indicated siRNAs/shRNAs. *N* = 21 to 26 individual wells (96-well plate) analyzed per condition from three independent replicates. Cell numbers are inside the bars. Bars represent means ± SEM. *P < 0.05 and ***P < 0.001; Kruskal-Wallis test.

To probe whether axonal PrP^PG14^ endoggresomes lead to neuronal dysfunction, we tested whether the intrinsic capacity of cultured hippocampal neurons expressing PrP^PG14^-mCh to intake calcium upon KCl-induced depolarization was compromised, as shown previously for cerebellar granule cultured neurons (*47*). Neurons transduced with PrP^WT^-mCh or PrP^PG14^-mCh adeno-associated virus (AAV-DJ) displayed high transduction efficiencies of 96 ± 2% and 97 ± 2% (means ± SEM), respectively, and endoggresome densities comparable to those observed in transfected axons (fig. S10). Single-cell calcium imaging of AAV-DJ-PrP^PG14^-mCh–transduced neurons preloaded with the calcium-sensitive dye Fluo-4 AM (acetoxymethyl esters) resulted in a defective calcium influx in response to KCl-induced depolarization compared to AAV-DJ-PrP^WT^-mCh–transduced neurons (Fig. 6, A and B). Reducing endoggresome formation via depletion of ARESTA component kinesin-1 (Fig. 4E) in neurons transduced with AAV-DJ-PrP^PG14^-mCh resulted in calcium influx dynamics comparable to those of neurons transduced with AAV-DJ-PrP^WT^-mCh (Fig. 6, A and B). Calcium intake was not altered in control *Kif5c^−/−^* neurons transduced with AAV-DJ-PrP^WT^-mCh (Fig. 6, C and D).

To further determine the effect of PrP^PG14^ endoggresomes, the cell viability of WT versus *Kif5c^−/−^* neurons or versus neurons with reduced Vps41 and Arl8b function expressing PrP^PG14^-mCh versus PrP^WT^-mCh was quantified. At 10 days after PrP^PG14^-mCh transfection, when endoggresomes were prominent in WT axons (fig. S6D), expression of PrP^PG14^-mCh resulted in a ~4.5-fold increase in neuronal death compared to nontransfected neurons or those expressing PrP^WT^-mCh (Fig. 6E). Notably, neuronal death 10 days after transfection was not significantly different in *Kif5c^−/−^* neurons expressing PrP^PG14^-mCh compared to those expressing PrP^WT^-mCh or not transfected, suggesting that reduced endoggresome formation in KIF5C-depleted axons prevented neuronal death. Moreover, neuronal death was decreased when neurons were treated with shRNAs against Arl8b and with siRNAs against Vps41 (Fig. 6F). These data indicate that endoggresomes are neurotoxic and decrease neuronal viability, and inhibiting their formation by reducing the function of ARESTA pathway components abrogates these defects.

## DISCUSSION

The accumulation of misfolded proteins inside axons is a prominent feature of neurodegenerative pathologies, including of prion diseases (*13*, *48*–*51*). By modeling the formation of intra-axonal mutant PrP aggregates involved in human prion disease (*24*), we uncovered endolysosomal pathways in mammalian neurons that sort post-Golgi mutant PrP vesicles into two divergent itineraries: one for immediate lysosomal degradation in the soma in a process resembling RESET (*21*, *22*), and the other toward the axon by an Arl8b/kinesin-1/HOPS ARESTA axis that ensures their axonal entry, fusion, and aggregation inside endolysosomes that we call endoggresomes (fig. S11). The opposing nature of these pathways appears to stem in large part from the highly polarized neuronal architecture, with an established highly degradative somatic compartment where mutant PrP is actively cleared versus an axonal compartment where ARESTA actively drives endoggresome formation and where limited lysosomal degradation sustains them. These distinct itineraries account for the enhanced vulnerability of axons versus the soma in generating aggregates and highlight the importance of investigating aggregate-driven mechanisms in neuronal systems. We posit that this soma versus axon compartmentalization also explains the differential susceptibility of neurons versus non-neuronal cells to the formation of aggregates and to aggregate-driven proteotoxicity that is characteristic of neurodegenerative disorders.

The discovery that mutant PrP undergoes degradation in the soma versus aggregation in the axon was unexpected because, in non-neuronal cells, essentially all misfolded PrP particles were shown to degrade via RESET, indicating that this pathway represents a bona fide and effective quality control and clearance mechanism for misfolded PrP (*22*). While the precise ratio of PrP^PG14^ particles undergoing immediate somatic degradation versus ARESTA-dependent aggregation in our neuronal system is unknown, our data show that expression of mutant PrP in neurons activates ER stress, followed by its delivery into lysosomes in the soma, suggesting that RESET-like degradative mechanisms are also at play in neurons. How the population of post-Golgi mutant PrP vesicles is selected for immediate degradation in the neuronal soma is unclear. A possible mechanism was shown for an artificial PrP mutant in non-neuronal cells, which triggers ER stress activation and traffics to the cell surface in a complex with ER chaperones and cargo receptors, and these associations appear to be critical tags for this itinerary toward degradation (*22*). Our observations showing that expression of mutant PrP robustly activates neuronal ER stress make this a plausible initial event that regulates the degradation via RESET, but this requires further investigation. Our data also show that a second population of post-Golgi mutant PrP vesicles skip somatic degradation but are instead funneled into the axon for aggregation by a regulated ARESTA process that depends on Arl8b recognition. Sorting of post-Golgi mutant PrP vesicles into the axon occurs by recruitment of kinesin-1/Vps41 (HOPS) by Arl8b, for association with ~80% of PrP^PG14^ vesicles. Arl8b is absent from RESET- and degradation-bound PrP^PG14^ vesicles, indicating that this complex could represent an axonal tag. Direct Golgi-to-axon cargo transport within the secretory pathway has been reported for other endosomal vesicles, including those carrying tropomyosin-related kinase B (TrkB) receptors (*52*). Moreover, Vps41 is known to be involved in direct Golgi-to-LE sorting (*53*), so this role appears conserved during interactions with mutant PrP vesicles. How Arl8b recognizes mutant PrP vesicles at the Golgi merits further investigation, but the mechanism could involve known Arl8b endosomal sorting regulators. A BORC [biogenesis of lysosome-related organelles complex (BLOC)-one related complex]-Arl8b complex that couples Arl8b to LEs has been shown to drive LE distribution uniquely toward the periphery of axons and not to dendrites (*45*, *54*), making it a candidate regulator of axonal entry of mutant PrP vesicles that merits further investigation. It is also plausible that other endolysosomal pathways could contribute to formation of aggregates inside axons, as the role of GTPases in the accumulation of mutant PrP is recognized in the literature. For example, genetic modulation of endosomal Rab11 results in the accumulation in the secretory pathway of mutant PrP^D177N/M128^ associated with fatal familial insomnia (FFI), a prion disease (*55*). In addition, overexpression of Rab9 inhibits PrP^Sc^ accumulation in neuronal cell lines, pointing to a role of LEs in prion conversion (*56*), and inhibition of MVB inhibition of MVB maturation via reduction of Rab7 activity resulted in the significant reduction of PrP^Sc^ levels, suggesting that MVBs could be sites of PrP^Sc^ conversion (*15*).

The identification of endoggresomes highlights the distinct nature of these structures from previously characterized intracellular cytosolic aggregate assemblies, including aggresomes, insoluble protein deposits (IPODs), and juxtanuclear quality control compartments (JUNQs) (*14*, *57*). In comparison to these structures, our data show that endoggresomes form inside membranes instead of the cytosol, depend on kinesin-based anterograde transport instead of dynein-based retrograde movement for their formation, form distally in axons and remain at distal sites away from perinuclear domains, are not clearly targeted for degradation, and impart toxicity rather than being cytoprotective. The presence of endoggresomes inside endomembrane structures raises the possibility that further dynamic sorting events may occur during their lifetime. It will be important in future studies to explore the possible maturation and dynamics of endoggresomes as they reside inside axons for longer periods of time, including subcellular events occurring within and around these pathophysiological hubs that could delineate local impairments to axonal function.

The presence of membrane-bound endoggresomes as neurotoxic structures in axons is consistent with previous observations showing intralysosomal accumulations of PrP^PG14^ in dystrophic swollen neurites in hippocampal and cerebellar granule cell layer neurons in transgenic mice (*58*). The generality of endoggresomes and ARESTA within the prionopathies and to other neurodegenerative pathologies requires further investigation. However, our analyses indicate that, at least in the case of PrP^D177N/M128^, the formation of intra-axonal aggregates also depends on kinesin-1C function, indicating that endolysosomal pathways might be a common aggregation mechanism of familial PrP mutations. Ultrastructural studies of human patient brains have shown the accumulation of other PrP mutants within dystrophic neurites inside endolysosomes (*13*, *48*–*51*, *59*), as well as in PrP^Sc^-infected cell lines, and in brains of rodent models of familial and sporadic human PrP^Sc^ disease, suggesting that ARESTA may be a generalized mechanism that accounts for decades-long observations of endolysosomal assemblies in dystrophic neurites in prion brains, including those caused by aggregation of misfolded WT PrP (*19*, *48*, *50*, *59*–*61*). Despite the presence within endolysosomes, mutant PrP has also been reported to form aggresomes in the cytosol of neurons, where they were targeted for clearance by ER-associated degradation (ERAD) (*12*), although whether these represent toxic structures is debated (*61*). However, extensive characterization of the trafficking and turnover of many misfolded GPI-anchored PrP mutants showed that they are normally poor ERAD substrates, are not dependent on proteasomal degradation, and exit the ER for degradation in lysosomes (*62*). Thus, it is possible that RESET-like degradation in the soma and endoggresome formation in axons are favored itineraries by misfolded PrP as primary pathways, but a shift toward aggresome formation may occur when and if RESET and eventually ERAD are overwhelmed or compromised following extensive and sustained ER stress. Once formed in axons, endoggresomes persist because of poor local axonal clearance and impaired retrograde transport toward the soma that would promote its degradation. Consistent with our observations, defects in lysosomal maturation and degradation, as well as in retrograde axonal transport, have been documented in neuronal cell lines persistently infected with prions (*63*), as well as in prion-infected mouse models of disease (*64*).

The generality of endoggresomes and ARESTA to other neurodegenerative pathologies is a tantalizing possibility given the reliance of tau, the amyloid precursor protein (APP)/amyloid beta (Ab), α-synuclein, and huntingtin on endosomal pathways for their processing and spread (*65*–*67*); thus, this prospect merits further investigation. Notably, enlarged endosomes represent a common endosomal “traffic jam” phenotype in Alzheimer’s disease (AD) and are considered among the earliest neuronal lesions identified thus far (*68*–*71*). Genome-wide association studies (GWAS), whole exome and genome sequencing data, and multiple human genetics analyses have identified endolysosomal genes as risk hubs associated with early pathophysiological changes in AD (*72*, *73*). It will be key to determine whether the ubiquitous enlarged endosomes observed in early-stage neurodegenerative pathologies and associated with cellular toxicity could be regulated by the ARESTA pathway. As genetic modulation of ARESTA components resulted in changes in endoggresome densities with measurable consequences to the function and viability of neurons, the identification of the Arl8b/kinesin-1/HOPS axis provides an antiaggregation target that can modulate neuronal dysfunction and delineate the differential vulnerability of axons to aggregate-induced pathologies.

## MATERIALS AND METHODS

### Mouse lines

Our mouse protocols were reviewed and approved by the Institutional Animal Care and Use Committee (IACUC) at The Scripps Research Institute (Scripps Research), and all colonies were maintained following the guidelines recommended by the Department of Animal Resources at Scripps Research. C57BL/6 WT mice were obtained from Charles River Laboratories. Kinesin-1C–null (*Kif5c*^−/−^) and KLC1-null (*Klc1*^−/−^) mice were generated as described previously (*30*). *Kif5c*^−/−^ mice were maintained as homozygotes and were backcrossed in the C57BL/6 background for at least six generations. *Klc1*^−/−^ mice were bred as heterozygotes to generate homozygous pups. The conditional homozygous kinesin-1B floxed mouse (*Kif5b^pflox/pflox^*) was generated as described previously (*38*).

### Primary neuronal cultures

Primary hippocampal neuronal cultures were prepared as described previously (*30*). Briefly, hippocampi were dissected from P0 to P2 mouse neonates in cold Hanks’ balanced salt solution (HBSS) (Gibco) supplemented with 0.08% d-glucose (Sigma-Aldrich), 0.17% Hepes, and 1% penicillin-streptomycin (Pen-Strep) (Gibco); filter-sterilized; and adjusted to pH 7.3. Dissected hippocampi were washed twice with cold HBSS (Gibco) and individually incubated at 37°C for 15 to 20 min in a sterile solution of 45 U of papain (Worthington), 0.01% deoxyribonuclease (DNase) (Sigma-Aldrich), 1 mg of dl-cysteine (Sigma-Aldrich), 1 mg of bovine serum albumin (BSA) (Sigma-Aldrich), and 25 mg of d-glucose (Sigma-Aldrich) in phosphate-buffered saline (PBS) (Gibco). Hippocampi were washed twice with Dulbecco’s modified Eagle’s medium (DMEM) (Gibco) supplemented with 10% fetal bovine serum (FBS) (Gibco) (preheated to 37°C) and disrupted by 10 to 12 cycles of aspiration through a micropipette tip. Dissociated neurons were then resuspended in warm DMEM (Gibco) documented with 10% FBS (Gibco) and plated in 24-well plates containing 12-mm glass coverslips pretreated with poly-l-lysine (50 μg/ml) (Sigma-Aldrich) in borate buffer [1.24 g of boric acid (Thermo Fisher Scientific) and 1.90 g of borax (Sigma-Aldrich) in 500 ml of cell culture–grade water, adjusted to pH 8.5, and filter-sterilized]. After 1 hour, the medium was replaced with Neurobasal-A medium (Gibco), supplemented with 2% B-27 (Gibco) and 0.25% GlutaMAX (Gibco). Primary neurons were maintained in an incubator at 37°C and in a 5.5% CO_2_ atmosphere.

For quantitative live imaging of axonal transport, hippocampal neurons were plated in microfluidic chambers or in regular 24-well glass coverslips as described previously (*30*). For calcium imaging, hippocampal neurons were plated directly onto #1.5 glass-bottom dishes (MatTek) precoated with poly-l-lysine (50 μg/ml) (Sigma-Aldrich) in borate buffer.

### Cell culture

N2a cells (American Type Culture Collection) were grown in DMEM (Gibco), supplemented with 10% FBS (Gibco) and 1% Pen-Strep (Gibco) in an incubator at 37°C and in a 5.5% CO_2_ atmosphere. Cells were passaged twice a week to low confluency using 0.05% trypsin-EDTA (Gibco).

### Design of DNA constructs

The MoPrP.Xho PrP^WT^-EGFP and MoPrP.Xho PrP^PG14^-EGFP constructs were a gift from D. Harris (Boston University) (*11*). All other constructs were generated using the In-Fusion HD Cloning Plus Kit (Clontech) for the final cloning of DNA fragments into the recipient vector. All polymerase chain reaction (PCR) amplifications were performed using the Phusion High-Fidelity DNA Polymerase (New England Biolabs). For the construction of MoPrP.Xho PrP^WT^-mCh and MoPrP.Xho PrP^PG14^-mCh constructs, EGFP was replaced with mCh using assembly PCR. Briefly, three individual PCRs were performed to amplify the (1) N terminus of PrP^WT^ or PrP^PG14^, (2) C terminus of PrP, and (3) mCh including 20– and 22–base pair overlaps to the 3′ of (1) and 5′ of (2), respectively.

(i) The N terminus of PrP was amplified using the following primers: P1 N terminus (forward): 5′-CTAGTGGTACCTCGAGATGGCGAACCTTGGCTAC-3′ and P2 N terminus (reverse): 5′-CATGGTGGCGACCGGTGGATCC-3′.

(ii) The C terminus of PrP was amplified using the following primers: P3 C terminus (forward): 5′-TCCGGACTCAGATCTCGAGCTCAAG-3′ and P4 C terminus (reverse): 5′-AGCAGGAAGGCTCGAGTCATCCCACGATCAGGAA-3′.

(iii) mCh was amplified using the following primers: P5 mCh (forward): 5′-ATCCACCGGTCGCCACCATGGTGAGCAAGGGCGAGGA-3′ and P6 mCh (reverse): 5′-GCTCGAGATCTGAGTCCGGACTTGTACAGCTCGTCCATGCCG-3′.

PCR products (1 to 3) were gel-purified and subsequently stitched together (1 ng each) using a PCR performed without primers. After 20 cycles, PCR was interrupted; primers P1 (forward) and P4 (reverse) (0.5 μM each) were added to specifically amplify the complete product. The reaction was supplemented with 100 μM dNTPs (deoxynucleotide triphosphates) and 0.5 mM MgCl_2_ and ran for 30 more cycles. Generation of MoPrP.Xho PrP^WT^-mTagBFP2 and MoPrP.Xho PrP^PG14^-mTagBFP2 constructs was done using the same assembly PCR strategy.

P5 mTagBFP2 (forward): 5′-ATCCACCGGTCGCCACCATGGTGTCTAAGGGCGAAGA-3′, and P6 mTagBFP2 (reverse): 5′-GCTCGAGATCTGAGTCCGGAATTAAGCTTGTGCCCCAGTT-3′.

Generation of the untagged MoPrP.Xho PrP^WT^ and MoPrP.Xho PrP^PG14^ was done using the same assembly PCR strategy.

(1) The N terminus of PrP was amplified using the following primers: P1 N terminus (forward): 5′-CTAGTGGTACCTCGAGATGGCGAACCTTGGCTAC-3′ and P2 N terminus (reverse): 5′-CATGGTGGCGACCGGTGGATCC-3′.

(2) The C terminus of PrP was amplified using the following primers: P3 C terminus (forward): 5′-CCGGTCGCCACCATGTCCGGACTCAGATCTCGAGCTCAAG-3′ and P4 C terminus (reverse): 5′-AGCAGGAAGGCTCGAGTCATCCCACGATCAGGAA-3′.

Generation of MoPrP.Xho PrP^WT^-PAmCh and MoPrP.Xho PrP^PG14^-PAmCh was done using the same assembly PCR strategy from PAmCh1-C1 (a gift from V. Verkhusha, Albert Einstein College of Medicine, New York) (*74*), which contains the following mutations: E26V/A58T/K69N/L84F/N99K/S148L/I165V/Q167P/L169V/I203R.

P5 PAmCh (forward): 5′-ATCCACCGGTCGCCACCATGGTGAGCAAGGGCGAGGA-3′ and P6 PAmCh (reverse): 5′-GCTCGAGATCTGAGTCCGGACTTGTACAGCTCGTCCATGCCG-3′.

To generate BBS-tagged constructs, MoPrP.Xho PrP^WT^-mCh-BBS and MoPrP.Xho PrP^PG14^-mCh-BBS, a 13–amino acid BBS tag (WRYYESSLEPYPD) was inserted at the Bsp EI site in the linker between mCh and C-terminal PrP sequence downstream of mCh. The following oligos were used to amplify the BBS sequence:

BBS (forward): 5′-CCGGATGGAGATACTACGAGAGCTCCCTGGAGCCCTACCCTGACT-3′ and BBS (reverse): 5′-TACCTCTATGATGCTCTCGAGGGACCTCGGGATGGGACTGAGGCC-3′.

The PCR products were annealed and cloned in PrP^WT^-mCh in a pcDNA3.1 plasmid and PrP^PG14^-mCh in a pcDNA3.1 plasmid. PrP^WT^-mCh-BBS and PrP^PG14^-mCh-BBS sequences were amplified by PCR and subcloned into the MoPrP.Xho vector. A similar strategy was used to generate the TCS-tagged constructs MoPrP.Xho PrP^WT^-mCh-TCS and MoPrP.Xho PrP^PG14^-mCh-TCS. The following oligos were used to amplify the six–amino acid TCS tag (LVPRGS) and to insert it at the Bsp EI site downstream of mCh:

TCS (forward): 5′-GCTGTACAAGTCCGGACTGGTGCCGCGCGGCAGCTCCGGACTCAGATCTC-3′ and TCS (reverse): 5′-GAGATCTGAGTCCGGAGCTGCCGCGCGGCACCAGTCCGGACTTGTACAGC-3′.

To generate the LAMP1-EGFP construct, red fluorescent protein (RFP) was replaced with EGFP in the plasmid LAMP1-RFP (a gift from E. Holzbaur, University of Pennsylvania). To generate SS-EGFP derived from NPY, the first 84 nucleotides corresponding to the SS of NPY were amplified from mouse brain complementary DNA (cDNA) and cloned between Bgl II and Bam HI sites in the pEGFP-N3 vector. To design EGFP-Rab5, EGFP-Rab7, and EGFP-Rab11a, the corresponding sequences were amplified from mouse brain cDNA and cloned between Xho I and Bam HI sites in the pEGFP-C1 vector (a gift from H. Lashuel, Swiss Federal Institute of Technology):

Rab5 (forward): 5′-TCTCGAGCTCAAGCTTTAATGGCTAATCGAGGAGCAACA-3′ and Rab5 (reverse): 5′-TAGATCCGGTGGATCCTCAGTTACTACAACACTGGCTTCTGG-3′, Rab7 (forward): 5′-TCTCGAGCTCAAGCTTATGACCTCTAGGAAGAAAGTGTTG-3′ and Rab7 (reverse): 5′-TAGATCCGGTGGATCCTCAACAACTGCAGCTTTCTG-3′, and Rab11a (forward): 5′-TCTCGAGCTCAAGCTTATGGGAACACGCGACGACGTA-3′ and Rab11a (reverse): 5′-TAGATCCGGTGGATCCGATGTTCTGACAGCACTGCACCTTT-3′.

T34N and Q75L mutations were introduced in pDEST47-Arl8b^WT^-GFP plasmid (Addgene, #67404) using the QuikChange II XL Site-Directed Mutagenesis Kit (Agilent). To generate untagged Arl8b constructs, the Arl8b coding sequence was cloned in the pBI-CMV3 bidirectional promoter vector (Clontech). The KLC1-TAP plasmid was a gift from L. S. B. Goldstein (University of California San Diego). 2xmCh-KIF5C was designed by subcloning 2xmCh from KIF5C(1–560)-2xmChEF(C) (Addgene, #61664) in pCDNA3.1. Then, a full-length KIF5C sequence was amplified from pGFP-KIF5C (a gift from M. Peckham, University of Leeds) and cloned downstream of 2xmCh. To generate the SKIP 1 to 186 construct, the sequence of the first 186 amino acids was cloned in pcDNA3.1 at the Hind III and Bam HI site.

SKIP 1 to 186 (forward): 5′-CTAGCGTTTAAACTTAAGCTTATGGAGCCGGGGGAGGTGAAG-3′ and SKIP 1 to 186 (reverse): 5′-CCACACTGGACTAGTGGATCCGACCGAGCTGGGAAGGCGGTC-3′.

### Transfections

Hippocampal neurons were transiently transfected using Lipofectamine 2000 (Thermo Fisher Scientific), following the manufacturer’s instructions. DNA and Lipofectamine were diluted in nonsupplemented Neurobasal-A medium (Gibco). For most experiments, neurons grown on 12-mm coverslips in 24-well plates were transfected using 2 μl of Lipofectamine and 0.4 or 0.8 μg of DNA per well. Otherwise, neurons were grown in microfluidic chambers and transfected using 1.2 μl of Lipofectamine and 0.5 μg of DNA per chamber. Medium was changed 1 hour after transfection.

N2a cells were transiently transfected using Lipofectamine 2000 (Thermo Fisher Scientific) following the manufacturer’s instructions. DNA and Lipofectamine were diluted in DMEM (Gibco). Experiments were performed in six-well plates, using 8 μl of Lipofectamine and 2 μg of DNA per well.

### Adenovirus and AAV transductions

Cre-recombinase adenovirus transduction (Ad5CMVCre; University of Iowa) was performed as described previously (*30*). Briefly, hippocampal neurons isolated from kinesin-1B conditional KO mice (*KifB^pflox/pflox^)* were treated at 6 days in vitro (DIV) with 0, 100, or 400 MOI of adenovirus, corresponding to 0, 5 × 10^6^, or 2 × 10^7^ plaque-forming units (PFUs), respectively. Neurons were incubated for 2 hours with the virus and then washed twice with Neurobasal-A medium (Gibco) containing 2% B-27 (Gibco) and 0.25% GlutaMAX (Gibco). Neurons were transfected with fluorescently labeled PrP constructs at 9 DIV and imaged at 11 DIV.

PrP^WT^-mCh and PrP^PG14^-mCh were cloned into the pAAV-hSyn-MCS backbone between the Bam HI and Eco RI sites (a gift from T. Golde, University of Florida).

Forward: 5′-CCGCGAGCTCGGATCCATGGCGAACCTTGGCTACTG-3′ and reverse: 5′-CTTCCTGATCGTGGGATGAGAATTCCTCGAGCAGC-3′.

AAV was made using the AAV-DJ Helper Free Packaging System (Cell Biolabs) using published protocols (*75*). Hippocampal neurons growing in 24-well plates were treated overnight with 4 μl of the viral stock at 7 DIV and imaged at 12 DIV.

### Knock down using shRNAs and siRNAs

To knock down Arl8b from neurons, the following 21-mer shRNA previously validated inserts were cloned separately in the pLKO.3G vector (Addgene, plasmid #14748): scrambled shRNA (CCTAAGGTTAAGTCGCCCTCG), Arl8b shRNA #1 (CCTCTCGAAATGAACTGCATA), and Arl8b shRNA #3 (CGAGGAGTCAATGCAATTGTT). Validation of shRNA constructs for the reduction of Arl8b was done by transfecting mouse N2a cells at 40% confluency with pLKO3.G-Arl8b shRNAs #1 and #2 either separately or together using Lipofectamine 2000 (Invitrogen). Dynein was knocked down by transfection of neurons with a previously validated DHC1 shRNA lentiviral construct (GTGATGCCATACGAGAGAA). A scrambled shRNA construct was used as control (GCACACGTATCGACGTATC) (*30*). Transfection rates were >80% and were ascertained by counts of GFP fluorescence on cell bodies. shRNAs were expressed for 48 or 72 hours before harvesting, lysed in radioimmunoprecipitation assay (RIPA) buffer, and evaluated by Western blot using rabbit anti-Arl8b (ProteinTech) and anti–α-tubulin as a loading control (Sigma-Aldrich). N2a cells were harvested at the same time points for reverse transcription PCR generation using an iScript cDNA Synthesis kit (Bio-Rad), and quantitative PCR (qPCR) was performed using the FastStart Universal SYBR Green Mastermix (Roche) to test for reduced Arl8b mRNA levels.

To knock down VPS41 from neurons, we used the ON-TARGETplus SMART pool siRNA from Dharmacon. VPS41 siRNA #1: CCAAAGGAACAUUAAACGA, VPS41 siRNA #2: GUUUGUACUGGCGGGAAA, VPS41 siRNA #3: GGAGAAGAAUUUCACGAGA, and VPS41 siRNA #4: UGACAUAAGUCUUCGCCCA. Nontargeting siRNA #1: UGGUUUACAUGUCGACUAA was used as a control.

### Live imaging microscopy

Live imaging of soma and axons of hippocampal neurons was done on 9- to 15-DIV neurons, 2 days after transfection, except otherwise indicated. In some experiments, fluorescently labeled PrP constructs were cotransfected with soluble EGFP or mCh constructs to visualize the neuronal morphology and facilitate the determination of axonal polarity. Coverslips (12 mm) were transferred and flipped onto 35-mm glass-bottom dishes (MatTek) containing 2 ml of Neurobasal-A medium (Gibco), containing 2% B-27 (Gibco) and 0.25% GlutaMAX (Gibco) medium. Neurons were imaged using a Nikon Ti-E Perfect Focus inverted microscope equipped with a total internal reflection fluorescence microscopy (TIRFM) setup, with an Andor iXon + DU897 EM camera, and a 100×/1.49 numerical aperture (NA) oil objective. Solid-state 405-, 488-, and 561-nm lasers were used for detecting mTagBFP2, GFP, and mCh, respectively. Lasers were positioned at varying angles for pseudo-TIRFM (pTIRFM) acquisition. Transfection rates were ~1%, which enabled imaging of individual neurons.

Time-lapse movies of fast vesicular transport in axons were 15 s long and collected at a frame rate of 10 frames/s (10 Hz). Movies of aggregate/endoggresome transport were 5 min (300 s) long and collected at a frame rate of 1 frame/s (1 Hz). Movies of LAMP1-EGFP vesicular transport were 1 min long and collected at a frame rate of 5 frames/s (5 Hz). For all images and movies acquired, the exposure time was set to 100 ms. Pixel size was 0.16 μm. Plates with cultured neurons were maintained at 37°C and 5.5% CO_2_ throughout the total imaging period. All axonal transport movies were taken in a central region of the axon, at least 150 μm away from both the soma and the axon tip.

For GFP/mCh axonal cotransport analyses, images were acquired using a Nikon near-simultaneous two-color LAMBDA 10-3 optical filter switch imaging setup. Exposure times were 75 ms, and 488/561-nm images were collected with a 50-ms delay. For live imaging of axonal transport, high-resolution imaging of vesicle cotransport was done using a high frame rate: 30 s long and at 5 frames/s (5 Hz). Low–frame rate cotransport movies were obtained for analyses of endoggresome transport: 5 min (300 s) long and collected at 1 frame/s (1 Hz). Time-lapse imaging was performed during 5 min at a frame rate of 5 frames/s.

### Fixation and immunofluorescence

Neurons were fixed with 4% paraformaldehyde (PFA; Electron Microscopy Services) containing 4% sucrose (Sigma-Aldrich) for 30 min at 37°C. Cells were washed once in 50 mM glycine in PBS and three more times in PBS. If applicable, cells were permeabilized by incubating the coverslips in 0.1% Triton X-100 (Sigma-Aldrich) in PBS for 8 to 10 min followed by three washes with PBS. Coverslips were incubated in blocking solution [10% immunoglobulin G (IgG)–free BSA and 5% donkey serum or goat serum in PBS; Jackson ImmunoResearch] for 30 min at room temperature (RT), followed by incubation with primary antibodies in blocking solution for 1 hour at RT or overnight at 4°C. After three washes in PBS, neurons were incubated with secondary antibodies in blocking solution for 1 hour at RT or overnight at 4°C. If applicable, neurons were counterstained with 300 nM 4′,6-diamidino-2-phenylindole (DAPI) (Thermo Fisher Scientific) for 5 min. After three washes in PBS, coverslips were washed once in H_2_O and mounted in ProLong Diamond antifade reagent (Thermo Fisher Scientific).

The following antibodies were used for immunofluorescence: mouse anti-KDEL (1:100; Santa Cruz Biotechnology), rabbit anti-GM130 (1:100; Abcam), goat anti-mouse CatB (1:40; R&D Systems), rabbit polyclonal IgG anti-mCh (1:100; Gentex), recombinant Fab anti-PrP (1:200; clone HuM-D13) (*76*), rabbit anti-Arl8b (1:200; ProteinTech), and mouse anti-Vps41 (1:100; clone D-12, Santa Cruz Biotechnology). The following antibodies were used for Western blot: rabbit anti-Arl8b (1:200; ProteinTech) and mouse anti–α-tubulin (1:500; Sigma-Aldrich).

### Correlative fluorescence and scanning electron microscopy

Hippocampal neurons were plated in microfluidic chambers and cotransfected at 8 DIV with PrP^PG14^-mCh and soluble EGFP. Two days after transfection, the positions of transfected neurons inside the microfluidic chamber were mapped using live fluorescence microscopy with a Nikon S Plan Fluor ELWD 20×/0.45 [infinity]/0-2 objective. Neurons were fixed in ice-cold 2.5% glutaraldehyde in 0.1 M Na cacodylate buffer (pH 7.3), and 1% trehalose was added before the removal of chamber to expose cells for washing with buffer. Neurons were fixed in 1% osmium tetroxide, washed thoroughly with distilled water, and dehydrated in a graded ethanol series. Samples were immersed in 100% hexamethyldisilazane (HMDS) for 3 min and allowed to dry before mounting on a stub with carbon tape. Samples were sputter-coated with iridium at 10 μA to a thickness of approximately 5 nm [Electron Microscopy Science (EMS) model 150T S]. The coverslips were then examined on a Hitachi S4300 scanning electron microscope (Hitachi High Technologies America Inc., Pleasanton, CA) at 5 kV with settings adjusted according to needs.

### Correlative fluorescence and S3EM

Hippocampal neurons were plated on 35-mm gridded #1.5 glass-bottom dish (Cellvis) pretreated with poly-l-lysine (50 μg/ml) (Sigma-Aldrich) in borate buffer and transfected at 8 DIV with PrP^PG14^-mCh using Lipofectamine 2000 (Thermo Fisher Scientific). Two days after transfection, neurons were first imaged using live fluorescence and phase-contrast microscopy with a Nikon S Plan Fluor ELWD 20×/0.45 [infinity]/0-2 objective to map the location of transfected neurons on the grid. PrP^PG14^-mCh endoggresome sites were imaged using superresolution radial fluctuation (SRRF) microscopy settings (*77*) (20 frames/s, 100 frames) with a pTIRFM setup, with an Andor iXon + DU897 EM camera, and a 100×/1.49 NA oil objective. Samples were fixed on the gridded culture dish while live imaging by carefully mixing an equal volume of 2× EM fixative (5% glutaraldehyde and 4% PFA in 0.1 M cacodylate buffer with 3 mM CaCl_2_) warmed to 37°C with 2 ml of Neurobasal-A medium (Gibco), containing 2% B-27 (Gibco) and 0.25% GlutaMAX (Gibco). Samples were immediately removed from the microscope, fixative medium solution was discarded, and cells were left in fresh ice-cold 1× fixative for 90 min and rinsed three times with ice-cold 0.1 M cacodylate buffer with 3 mM CaCl_2_. Coverslips were postfixed and stained with 1.5% reduced osmium for 35 min, rinsed five times with MilliQ water, and stained again with 1% aqueous uranyl acetate for 1 hour at RT before serial dehydration with graded solutions of ice-cold ethanol in water. Samples were then fully dehydrated in two 10-min rinses of anhydrous ethanol at RT before infiltration with Durcupan resin. After 3:1, 1:1, and 1:3 ethanol-resin 2-hour infiltration steps, the samples were infiltrated with pure resin for 2 hours before another change of fresh pure resin and left overnight at RT. A final change of fresh resin was performed, and infiltrated samples were polymerized for 48 hours at 65°C. Coverslips were dissolved by immersion in concentrated hydrofluoric acid. Correlative light-electron microscopy was achieved using laser-branded fiducials in a thinly embedded sample, as previously described (*78*). Briefly, regions of interest (ROIs) that included SRRF-imaged PrP^PG14^-mCh axon segments were identified by their grid positions and dark osmium staining. ROIs were marked using the cutting laser of a Zeiss PALM laser cutting microscope to provide orientation and fiducial guides for further trimming and ultramicrotomy. ROIs were carefully identified under a dissecting microscope and excised from the coverslip using a jeweler saw and a scalpel, with careful thought given to the future block-face orientation. The small (1 mm by 2 mm by 2mm) sample block was glued to a blank resin block such that the ROIs were orthogonal to the cutting plane and approximately 100 μm from the cutting surface. Laser marks on the block face helped to identify the location of the ROI using the ultramicrotome optics. The block was carefully trimmed using a 90° diamond trimming knife (Diatome). About 200 serial sections were collected in total over four chips.

The chips were mounted on aluminum stubs using double-sided carbon sticky tape and loaded into a Zeiss Sigma VP scanning electron microscope. Chip mapping and the imaging of serial sections were facilitated by SmartSEM (Zeiss) and Atlas 5 (Fibics) software packages. Maps of all of the serial sections on the silicon chips were collected at 500 nm per pixel. Laser marks could be identified in the resin boundary at low magnification, demarcating the ROI in each section. The ROI of the axon segment containing the PrP^PG14^-mCh endoggresomes was identified and captured across 40 serial-section scanning electron micrographs. ROIs were imaged at 2 nm per pixel using an electron backscatter detector (Gatan). In high-vacuum mode, a 3-keV beam in high-current mode with a 30-μm aperture at a working distance of 9 mm was found to produce signal from which we could resolve membrane and organelle (e.g., microtubule) ultrastructure. Sections were aligned using Photoshop (Adobe) and elastic alignment functions embedded in Fiji [National Institutes of Health (NIH)]. A central orthogonal projection (i.e., *x*-*z*) of the S3EM data was used to correlate the SRRF-processed data and produce overlay. Sections were sampled from throughout the aggregate and nonaggregate sites with reference to the correlated volume.

### Hippocampal neuron live assays with drug treatments and fluorescent dyes

To retain PrP^PG14^ in the ER, BFA (BioLegend) was applied at 5 μg/ml at the same time as PrP^WT^-mCh or PrP^PG14^-mCh transfections and incubated for 2 to 6 hours. Neurons were washed two to three times with Neurobasal-A medium (Gibco) before fixation.

For characterization of PrP^PG14^ at the cell surface of the soma or axons, neurons grown in 24-well plates were transfected with a PrP^PG14^-mCh-BBS construct for 1 hour. Neurons were treated with BTX-a647 (Molecular Probes) by applying it to the medium and as described previously (*32*). Briefly, we pretreated neurons for 1 hour with 100 μM tubocurarine hydrochloride (Sigma-Aldrich) to eliminate nonspecific binding to other receptors (*32*). Neurons were then incubated with BTX-a647 (7 μg/ml) in the medium, during indicated times and at indicated temperatures. To inhibit clathrin-mediated endocytosis, 80 μM dynasore (Sigma-Aldrich) was added 30 min before BTX-a647 treatment and kept during the labeling. DMSO was used as a control. Internalized PrP^PG14^ particles were defined as PrP^PG14^-mCh-BBS particles that were both positively labeled with BTX-a647 and actively transporting within axons. Quantification of internalization in the presence or absence of dynasore was performed by measuring the proportion of BTX-a647–positive particles that were actively transporting.

To characterize PrP axonal degradation in axons, neurons transfected with PrP^WT^-mCh-BBS or PrP^PG14^-mCh-BBS were incubated with BTX-a647 for 10 min, followed by a wash-off. Axons where imaged at 1 and 24 hours after BTX-a647 treatment. PrP axonal degradation was determined by drawing ROIs around the axons and by image analysis following integration of the total gray value.

PrP surface expression pattern in axons was analyzed by incubating neurons with BTX-a647 during 10 min at 4°C before fixation. LysoTracker Green (Molecular Probes) was incubated at 50 nM, 1 to 2 hours before live imaging. Magic Red from the Cathepsin B Assay (ImmunoChemistry Technologies) was incubated for 1 to 2 hours before live imaging. DMSO stock (260×) of Magic Red dye was diluted in sterile diH_2_O, and 10 μl was applied onto neurons in each well containing 250 μl of Neurobasal-A medium (Gibco). BafA1 (Sigma-Aldrich) was incubated at 10 nM during 6 hours. Longer incubation times affected neuronal survival. DMSO was used as a control.

For the characterization of PrP^PG14^ lysosomal degradation in the soma, hippocampal neurons were transfected with PrP^PG14^-mCh or PrP^WT^-mCh. One day after transfection, neurons were treated with 10 nM BafA1 for 6 hours and 50 nM LysoTracker Green for 1 hour before live imaging of the soma. PrP accumulation was determined by drawing ROIs around the soma and integration of the total gray value.

For characterization of PrP^PG14^ cleavage at the cell surface, hippocampal neurons were transfected with PrP^PG14^-mCh-TCS or PrP^PG14^-mCh. Neurons were incubated with or without thrombin protease (5 U/ml) (Sigma-Aldrich) 2 hours after transfection. This treatment was maintained for 48 hours. We did not observe any effect of thrombin treatment (5 U/ml) on neuronal survival.

For characterization of (in)dependence between direct and indirect ARESTA, hippocampal neurons were transfected with PrP^PG14^-mCh-TCS and Arl8b^WT^-GFP. Neurons were incubated with or without thrombin protease (5 U/ml) (Sigma-Aldrich) 24 hours after transfection. This treatment was maintained for 24 hours. We did not observe any effect of thrombin treatment (5 U/ml) on neuronal survival.

### Photoactivation of PrP^PG14^-PAmCh

Neurons were cotransfected with EGFP-Rab7 and PrP^PG14^-PAmCh. Two days after transfection, PrP^PG14^-PAmCh was photoactivated using a 405-nm laser burst pointed at the cell body (40% laser power, 3-s exposure), and neurons were imaged using live fluorescence microscopy. The fate of single photoactivated particles and their delivery into axons was followed for 60 min by taking 5 × 5 stitching images every 5 min.

### PK resistance assay

N2a cells were transfected with PrP^WT^-mCh or PrP^PG14^-mCh (pcDNA3.1 vector) and lysed 48 hours after transfection in PBS containing 0.5% NP-40 (Sigma-Aldrich), 0.5% sodium deoxycholate (Sigma-Aldrich), 0.2% sarkosyl (Sigma-Aldrich), and 0.5% Triton X-100. Proteins in 500 μg of lysate were digested using PK (0, 0.05, 0.1, 0.5, 1, or 5 μg/ml; Thermo Fisher Scientific) for 30 min at 37°C. The reaction was blocked by adding freshly prepared phenylmethylsulfonyl fluoride (PMSF) (0.8 mg/ml) (TCA; Sigma-Aldrich). The equivalent of 20 μg of each sample was analyzed by immunoblotting using an anti-PrP–specific antibody (D13) (a gift from D. Burton, Scripps Research). Samples corresponding to 1 and 5 μg/ml of PK were also precipitated using trichloroacetic acid (TCA; Sigma-Aldrich) to concentrate the proteins. The equivalent of 120 μg of each sample was analyzed by immunoblotting.

The PK assay of the sucrose gradient–floated fraction was done on 20 μg of an 8/35% sucrose-floated vesicle fraction that was digested using PK (0, 0.5, 1, 2, or 5 μg/ml) for 30 min at 37°C. The digestion reaction was blocked by addition of freshly prepared PMSF (0.8 mg/ml) (Sigma-Aldrich). The sample was analyzed by immunoblotting with Fab anti-PrP (1:200; clone Hum-D13).

### Detergent insolubility assay

A detergent insolubility assay was used following previously established protocols (*60*). Briefly, N2a cells were transfected with PrP^WT^-mCh or PrP^PG14^-mCh constructs using Lipofectamine 2000 (Thermo Fisher Scientific). Two days after transfection, membrane-bound vesicles were isolated in a sucrose gradient vesicle floatation assay. Vesicles in the 8/35% sucrose fraction were lysed in a buffer containing 150 mM NaCl, 50 mM tris-HCl (pH 7.5), 0.5% Triton X-100, and 0.5% sodium deoxycholate, supplemented with protease inhibitors [pepstatin and leupeptin (1 mg/ml), 0.5 mM phenylmethylsulfonyl fluoride, and 2 mM EDTA). Lysates were centrifuged for 5 min at 16,000*g* to remove cellular debris, followed by ultracentrifugation at 265,000*g* for 40 min to pellet detergent-insoluble protein. Fifteen micrograms of both pellet and supernatant fraction was analyzed by immunoblotting using Fab anti-PrP (1:200; clone Hum-D13).

### SDS-PAGE and immunoblotting

Neuronal lysates were resuspended in Laemmli buffer and resolved by SDS–polyacrylamide gel electrophoresis (SDS-PAGE) in 8 to 12% polyacrylamide gels. Proteins were electrotransferred onto polyvinylidene difluoride (PVDF) membranes. Membranes were blocked for 30 min at RT in tris-buffered saline (TBS) containing 0.1% Tween 20 and 5% dry milk. They were incubated overnight at 4°C with primary antibodies diluted in TBS-Tween-milk. After six washes in TBS-Tween, the membranes were further incubated for 1 hour at RT with fluorescent secondary antibodies (LI-COR) diluted in TBS-Tween-milk. After six more washes in TBS-Tween, the membranes were imaged using LI-COR Odyssey imaging system using recommended antibodies.

### Sucrose gradient vesicle flotations

N2a cells grown in 150 mm by 20 mm dishes were transfected with PrP^WT^-mCh or PrP^PG14^-mCh constructs using Lipofectamine 2000 (Thermo Fisher Scientific). Cells were washed twice with PBS (Gibco) and harvested from each 150-mm dish using 10 ml of PBS. Cells were harvested and resuspended in 0.5 ml of a solution composed of 8% (w/v) sucrose (Sigma-Aldrich) in distilled water, 3 mM imidazole (Sigma-Aldrich), and 1× cOmplete Protease Inhibitor (Millipore-Sigma). Resuspended cells were homogenized using a 25-gauge syringe needle attached to a 1-ml syringe (Sigma-Aldrich); samples were homogenized by 20 passages. A 100-μl fraction of the resulting homogenate was collected (fraction 1), and the remainder was centrifuged at 3000*g* using a Sorvall RT7 Plus benchtop centrifuge (Thermo Fisher Scientific). The supernatant was collected, and the pellet was again resuspended in a minimal volume of 8% sucrose solution and centrifuged as above. The two rounds of supernatant fractions were pooled to constitute the post-nuclear supernatant (PNS) (fraction 2). The PNS in 8% (w/v) sucrose was mixed with a solution of 70% (w/v) sucrose in distilled water, 3 mM imidazole, and 1× cOmplete Protease Inhibitor to constitute a ~40% sucrose solution containing the PNS.

The PNS was bottom-loaded on a sucrose step gradient consisting of 35 and 8% sucrose. After centrifugation at 41,500 rpm for 2 hours at 4°C, on an Optima LE-90K SW50.1 rotor (Beckman Coulter), fractions were collected, including the 8/35% (8/35) sucrose fraction–containing membrane-bound floated vesicles. Protein concentration of fractions were measured using a Pierce BCA protein assay kit (Thermo Fisher Scientific), run on SDS-PAGE, and analyzed by Western blot using Fab anti-PrP (1:200; clone Hum-D13), anti-mCh polyclonal antibody raised in rabbit (1:1000) (Genetex), or anti-Rab7 monoclonal antibody raised in rabbit (1:1000; clone D95F2 XP, Cell Signaling Technology).

### Calcium imaging

Hippocampal cultures from WT (C57BL6) or *Kif5c^−/−^* mice were plated directly on #1.5 glass-bottom dishes (MatTek), at a density of 8 to 15 neurons/420 μm^2^ of field of view. Cultures were loaded with 2 μM Fluo-4 AM (Thermo Fisher Scientific) in Neurobasal-A medium supplemented with B-27 without phenol red for 30 min at 37°C in a 5.5% CO_2_ atmosphere, as recommended by an Invitrogen protocol. Cultures were washed three times and incubated for another 30 min in the B-27–supplemented Neurobasal-A without phenol red before imaging to allow complete de-esterification of intracellular AM esters. Before and during imaging, a calcium imaging buffer [140 mM NaCl, 5 mM KCl, 2 mM CaCl_2_, 0.8 mM MgCl_2_, 10 mM glucose, and 10 mM Hepes (pH 7.4)] was applied. Neurons were imaged using a Nikon S Plan Fluor ELWD 20×/0.45 [infinity]/0-2 objective in a temperature-controlled enclosure set to 37°C. Epifluorescence excitation was provided by a metal halide–doped mercury arc lamp (Nikon Intensilight). High temporal-resolution images (5 frames/s) were taken for 1 min with an electron multiplying charge coupled device camera (EM-CCD; Andor iXon3 897). Fluo-4 AM fluorescence were collected using Chroma filters (ET-GFP [ET470/40x T495lpxr, ET525/50m]). Depolarization was induced by applying KCl to a final concentration of 30 mM at the 30-s time point during acquisition. Still images of PrP^WT^-mCh or PrP^PG14^-mCh of the same region were collected using ET-DsRed [ET545/30x, T570lp, ET620/60m] Chroma filter.

### Cell death assay and imaging

ReadyProbes Cell Viability Imaging Kit (Blue/Green) (Thermo Fisher Scientific) was used to quantify cell death. Briefly, two drops of NucBlue Live reagent (Hoechst 33342) and two drops of NucGreen Dead reagent were added onto 10- to 14-DIV hippocampal neurons grown on the coverslips containing 1 ml of Neurobasal-A medium (Gibco), supplemented with 2% B-27 (Gibco) and 0.25% GlutaMAX (Gibco). Neurons were incubated at 37°C for 30 min before fixation with 4% PFA (Electron Microscopy Services) containing 4% sucrose (Sigma-Aldrich) for 30 min at 37°C. Cells were washed once in 50 mM glycine in PBS and three more times in PBS before mounting in ProLong Diamond antifade reagent (Thermo Fisher Scientific). Fixed neurons were imaged using a Nikon S Plan Fluor ELWD 20×/0.45 [infinity]/0-2 objective. Epifluorescence excitation was provided by a metal halide–doped mercury arc lamp (Nikon Intensilight). Blue and green nuclei staining were collected using UV-2E/C [AT350/50x, T400lp, ET460/50m] and ET-GFP [ET470/40x, T495lpxr, ET525/50m] Chroma filters, respectively. PrP^WT^-mCh or PrP^PG14^-mCh fluorescence were collected using the ET-DsRed [ET545/30x, T570lp, ET620/60m] Chroma filter.

### Quantification and statistical analysis

#### Neuronal soma image analysis

All images of neuronal soma were processed in ImageJ (NIH). Rolling-ball background fluorescence correction was applied to all images. 3D deconvolution was applied using synthetic Gaussian point spread function (PSF) and Richardson-Lucy algorithms in DeconvolutionLab2 (*79*). All images and line scan fluorescence intensity graphs were displayed as maximum intensity projection.

#### Analysis of axonal transport dynamics from time-lapse movies

Axonal transport analysis was performed using the custom-made and previously validated KymoAnalyzer ImageJ package of macros (*29*). KymoAnalyzer is freely available for download from our laboratory website (www.encalada.scripps.edu/kymoanalyzer), and a detailed description of the software and the definition of all parameters calculated are available in the website and in our publication (*29*). Briefly, kymographs were generated from time-lapse movies. Particle trajectories were manually assigned from the kymograph images. Track and segment-related parameters were automatically calculated by KymoAnalyzer.

#### Colocalization and cotransport quantifications

We determined colocalization by merging line scan intensity profiles of different channels in the same graph and quantifying the average number of peaks that overlapped between different channels. For cotransport analyses, individual and merged color kymographs were generated, and quantification of the average number of tracks that overlapped between each pair of vesicular cargos was analyzed. Vesicle tracks were sorted in anterograde, retrograde, and stationary populations, or as indicated for individually analyzed parameters. Endoggresome tracks were sorted in mobile and stationary populations or as indicated for individually analyzed parameters.

#### Definition of PrP^PG14^ vesicles versus aggregates/endoggresomes

We defined PrP^PG14^ endoggresomes/aggregates by (i) their biochemical profile, a definition that we used in biochemical assays, or by (ii) their fluorescence intensity profile in imaging assays, when PrP^PG14^ was tagged with a fluorescence protein (PrP^PG14^-mCh or PrP^PG14^-EGFP), or untagged, as recognized with antibodies against PrP, a definition that we used for axonal transport and axonal density analyses. First, biochemically, PrP^PG14^ endoggresomes were defined as done previously (*11*), as PrP^PG14^ protein that is recognized on an SDS-PAGE gel compared to PrP^C/WT^ by their (i) detergent insolubility and (ii) limited PK digestion at PK concentrations below 1 to 2 μg/ml compared to PrP^WT^ (*25*, *27*, *80*). Second, in fluorescence imaging assays for axonal transport analyses, fluorescently tagged (mCh or EGFP) PrP^PG14^ endoggresomes/aggregates were recognized as those PrP^PG14^ particles whose transport was slow (<0.5 μm/s) in movies taken at 1 Hz for 5 min. At this temporal resolution, PrP^PG14^-mCh or PrP^PG14^-EGFP vesicles were too faint and moved too fast (0.5 to 4 μm/s) to be tracked accurately. Instead, PrP^PG14^-mCh– or PrP^PG14^-EGFP–tagged vesicles were identified as those PrP^PG14^ particles actively moving at speeds that resembled those of PrP^WT^-mCh or PrP^WT^-EGFP vesicles (between 0.5 and 3.0 μm/s) when imaged at 10 Hz (imaged for 15 s). To quantify densities of PrP^PG14^ endoggresomes/aggregates in fixed axons, still images were taken of axons or neurons transfected with fluorescently labeled PrP^PG14^ constructs (mCh or EGFP) or transfected with untagged PrP^PG14^ and stained by antibodies against PrP. Quantification of the maximal gray intensities of five small PrP^PG14^ vesicle puncta was done for each image analyzed, after subtracting the fluorescence background using the Brightness and Contrast tool of ImageJ. PrP^PG14^ endoggresome/aggregates were defined as particles that displayed maximal gray fluorescence intensities 1.5 times above that of the average maximal gray fluorescence intensities of five PrP^PG14^ vesicles in the same axon. The particles still present in the image after thresholding were considered as endoggresomes. Axon length was measured using the polyline tool of ImageJ. Endoggresome density was expressed as the average number of aggregates per 100 μm of the axon length.

#### Calculation of densities of LysoTracker-positive particles

For LysoTracker-positive vesicle densities, we measured the axon length using the polyline tool of ImageJ and quantified all the particles labeled by LysoTracker Green along this line. Densities were plotted per 100 μm of the axon length.

#### ERSE-mCh and Vps41 fluorescence quantification

Neurons were cotransfected with the pEGFP-C1, MoPrP.Xho PrP^WT^-EGFP or MoPrP.Xho PrP^PG14^-EGFP, and the transcriptional ER stress reporter ERSE-mCh (a gift from L. Plate, Vanderbilt University) (*46*). Increases in the activity of ER stress pathways activate ERSE, and soluble mCh is synthesized. Total fluorescence intensities (gray values; arbitrary unit) of ERSE-mCh and Vps41 were quantified using ImageJ, using circular ROIs of 30 μm in diameter centered around the soma. The polyline tool was used to draw ROIs spanning the entire axonal length.

#### Calcium imaging analysis

ROIs were marked around the soma of neurons expressing PrP^WT^-mCh or PrP^PG14^-mCh, and we measured the average fluorescence for each ROI at a given time point. The average Fluo-4 AM fluorescence over time was collected using an ImageJ Time Series Analyzer V3 plugin and normalized to Δ*F*/*F* by subtracting the fluorescence intensity of each time point (*F*) to an average fluorescence before addition of KCl (*F*_0_) and divided by *F*. Rolling-ball background fluorescence correction was applied to all images before fluorescence intensity analysis.

#### Cell death quantitation

All image processing was done in ImageJ. The total number of NucBlue Live–positive nuclei (blue channel) was determined as follows: Background fluorescence was corrected on each image by applying a Gaussian blur filter with a radius of 12 to a duplicate of each image and subtracting this filtered image from the original. An automatic threshold was applied to the resulting images using the Li algorithm followed by a watershed filter, both in ImageJ. Total nuclei were counted using a minimum size threshold of 15 and circularity of 0.10. The total amount of NucGreen Dead–positive nuclei (green channel) were determined using the same method, now using a Gaussian blur filter with a radius of 10, thresholding using the Yen algorithm available in ImageJ, and nuclei determination using a minimum size threshold of 15 and circularity of 0.10.

#### Generation of graphs and figures

Graphs of average values were generated using Microsoft Excel. Cumulative frequency graphs were generated using the CDF function in MATLAB (MathWorks). Box plots were generated using BoxPlotR, an application in the shiny package from RStudio (http://shiny.chemgrid.org/boxplotr/). Figures were drawn in Adobe Illustrator.

#### Statistical analyses

All the transport parameters measured in this study were first tested for normality using the MATLAB Lilliefors test. For parameters following a normal distribution, we performed Student’s *t* test. For non-normal parameters, we performed a permutation *t* test (rndttest function in MATLAB). For all parameters, differences in medians were also checked using the Wilcoxon rank sum test (rnk function in MATLAB). The nonparametric Kolmogorov-Smirnov test was used to evaluate the equality of two sample distributions (in cumulative distribution functions). Most of the parameters are presented as means ± SEM. Multiple comparison corrections were used where appropriate. Details about each parameter, including definition of center, value and definition of *n*, statistical test used, and *P* values, can be found in specific figures and/or in the figure legends.
