# Supplementary material for: Endosomal sorting drives the formation of axonal prion protein endoggresomes
Source: Sci Adv. 2021 Dec 22;7(52):eabg3693. doi: 10.1126/sciadv.abg3693 (PMC8694590; doi:10.1126/sciadv.abg3693)
Supplement: Supplementary file 1 — Figs. S1 to S11 Legends for movies S1 to S3 [file sciadv.abg3693_sm.pdf]

Science Advances
AAAS

Supplementary Materials for

Endosomal sorting drives the formation of axonal prion protein endoggresomes

Romain Chassefeyre, Tai Chaiamarit, Adriaan Verhelle, Sammy Weiser Novak, Leonardo R. Andrade, André D. G. Leitão, Uri Manor, Sandra E. Encalada*

*Corresponding author. Email: encalada@scripps.edu

Published 22 December 2021, *Sci. Adv.* **7**, eabg3693 (2021)
DOI: 10.1126/sciadv.abg3693

The PDF file includes:

Figs. S1 to S11
Legends for movies S1 to S3

Other Supplementary Material for this manuscript includes the following:

Movies S1 to S3

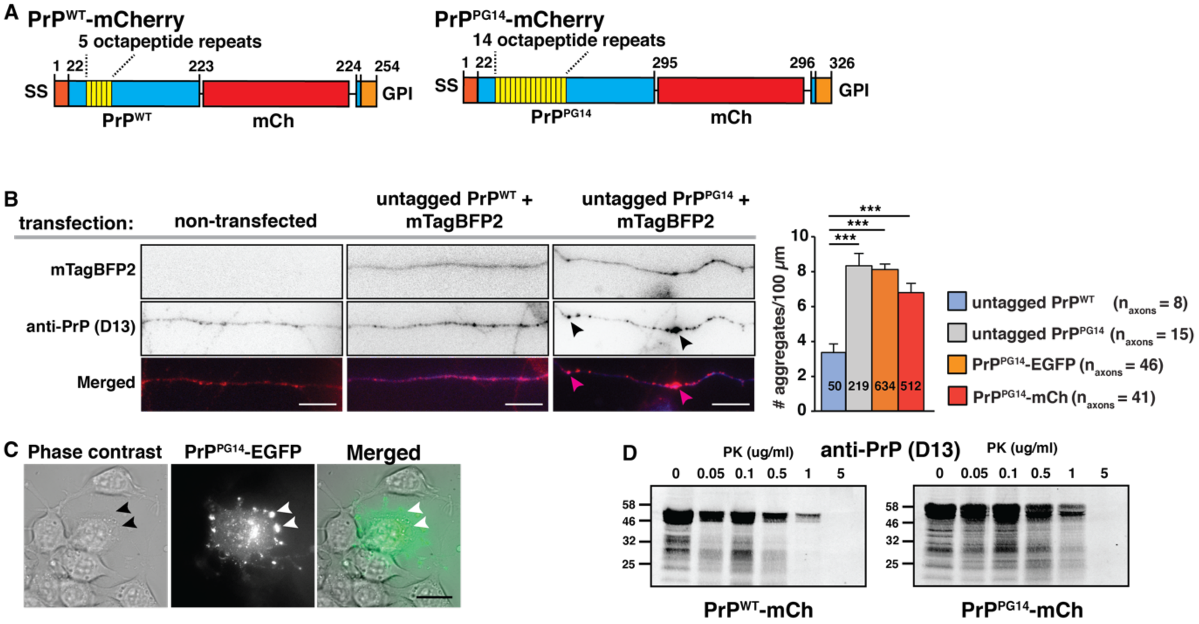

Fig. S1. Misfolded PrP^{PG14} forms aggregates inside axons. (A) Schematic of PrP^{WT}-mCh and PrP^{PG14}-mCh constructs. SS: signal sequence, GPI: GPI anchor. **(B)** Images of non-transfected axons and those co-expressing untagged PrP^{WT} or PrP^{PG14} with soluble mTagBFP2 (left) and aggregate quantification (right). Arrowheads point to aggregates. Scale bar = 10 µm. Bars represent means ± SEM. ***p<0.001, Kruskal- Wallis test. Aggregate numbers are shown inside bars. **(C)** Phase contrast and fluorescence images of a differentiated neuroblastoma (Neuro2a) cell expressing PrP^{PG14}-EGFP. Scale bar = 20 µm. **(D)** Representative Western blots showing of partial Proteinase K (PK) resistance by PrP^{PG14} aggregates from N2a cell lysates. Number of independent biological replicates = 3.

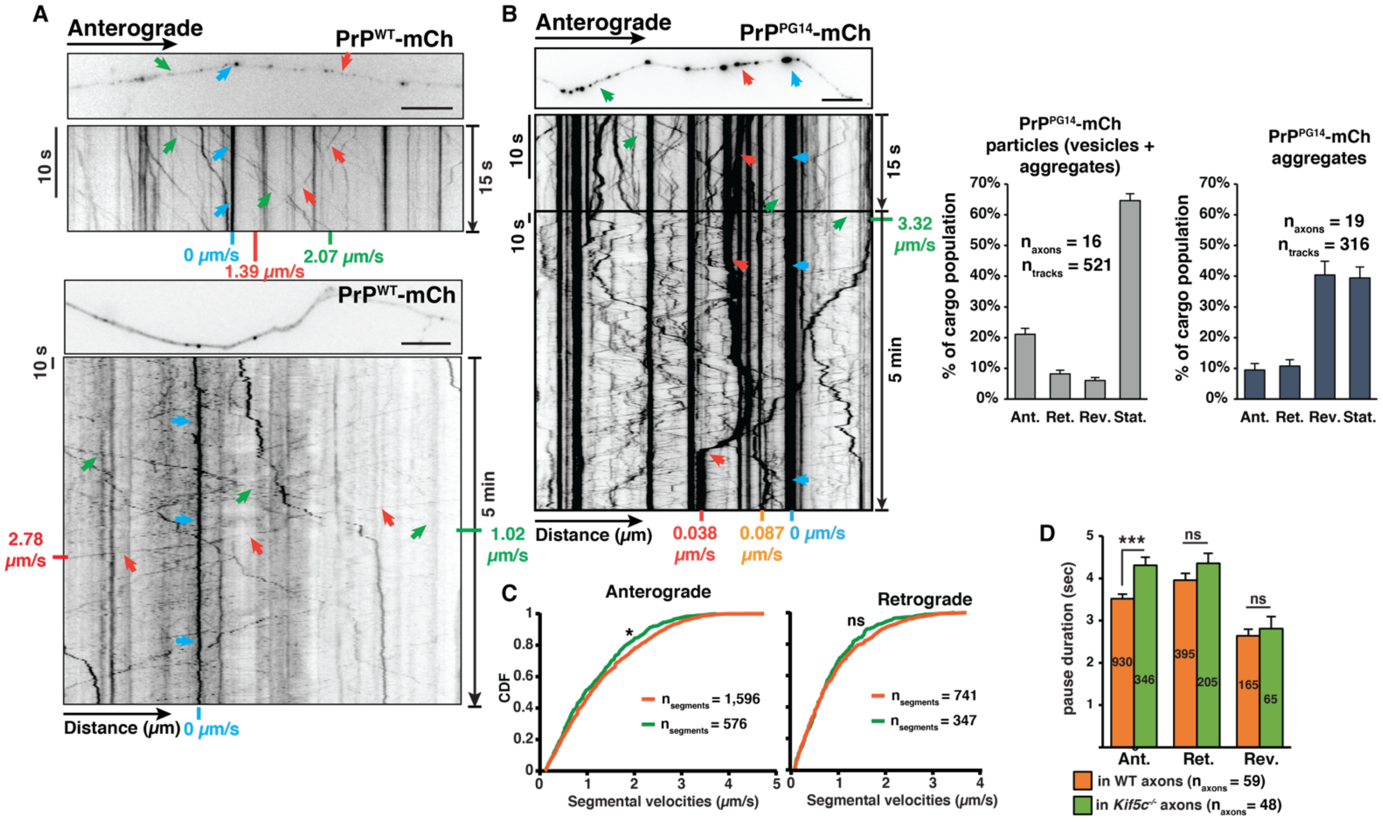

Fig. S2. PrP^{PG14} vesicles are transported in axons by kinesin-1. (**A**) First frames (top panels of each set) of time-lapse movies of axons expressing PrP^{WT}-mCh and corresponding kymographs (bottom panels of each set) at two time-scales. Arrows point to vesicles moving in the anterograde (green) or retrograde (red) directions, or that are stationary (blue). Scale bars = 10 µm. (**B**) First frame of time-lapse movie of an axon expressing PrP^{PG14}-mCh (top), and corresponding kymographs (middle-bottom) at two time-scales (left). Arrows point to vesicles or aggregates moving in the anterograde (green) or retrograde (red) directions, or that are stationary (blue). Scale bar = 10 µm. Population breakdown of PrP^{PG14}-mCh vesicles and aggregates (right). Bars represent means ± SEM. Ant. = anterograde, Ret. = retrograde, Rev. = reversals, Stat. = stationary. (**C**) Cumulative distribution frequency (CDF) of anterograde and retrograde

segmental PrP^{PG14}-EGFP vesicle velocities. *p<0.05, Kolmogorov-Smirnov test. (**D**) Pause duration of anterograde, retrograde and reversing PrP^{PG14}-EGFP vesicles. Numbers of vesicles are shown inside bars. Bars represent means ± SEM. ***p<0.001, ns = not significant, Student's t-test. Ant. = anterograde, Ret. = retrograde, Rev. = reversals.

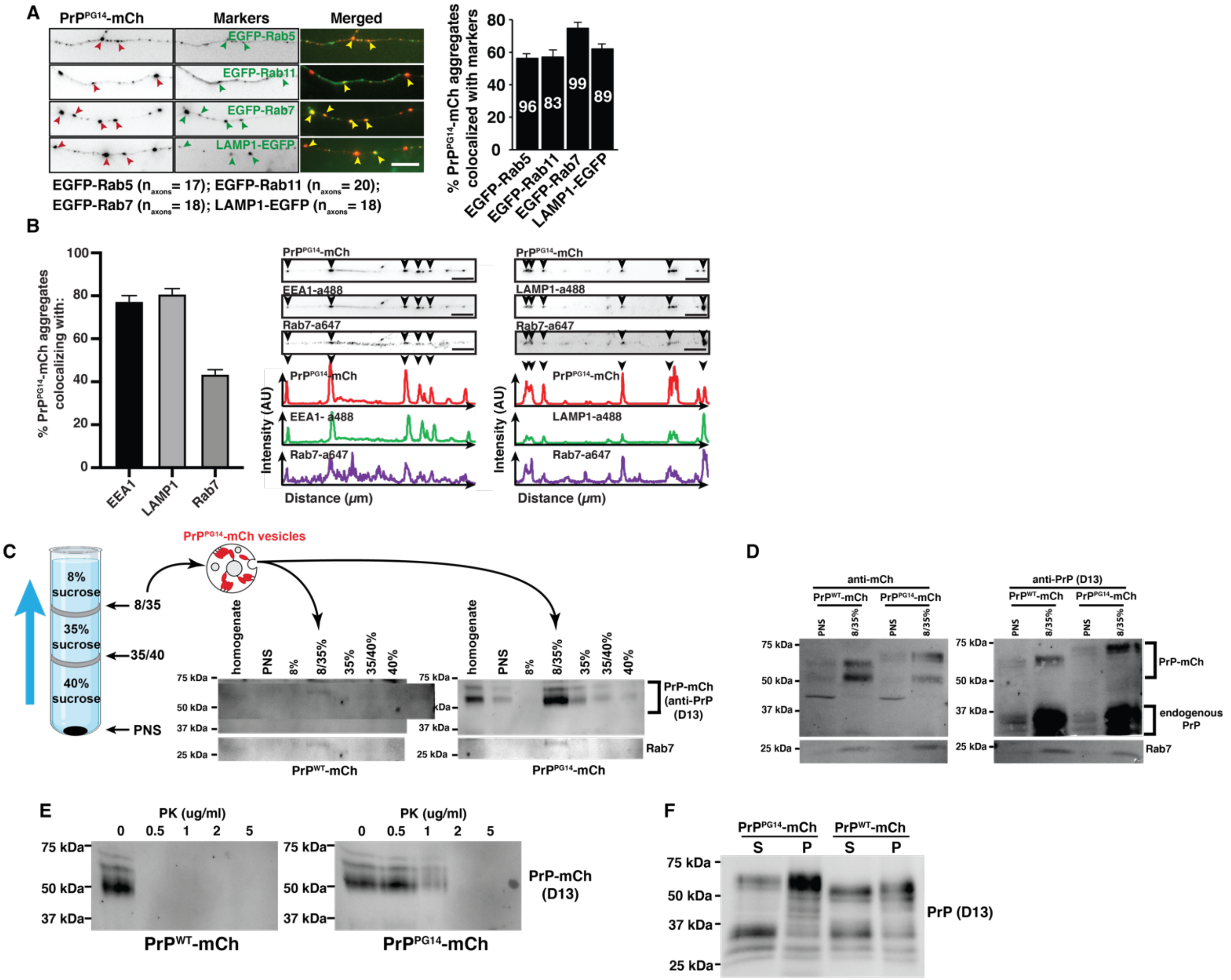

Fig. S3. Misfolded PrP^{PG14} resides in endolysosomal compartments. (**A**) Representative images of axons 2 days post-co-transfection with PrP^{PG14}-mCh and markers for EGFP-labeled Rab5, Rab11, Rab7, and LAMP1 (left). Arrowheads point to colocalizing aggregates. Scale bar = 10 µm. Quantitation of aggregate densities (right). Numbers of aggregates shown inside bars. Bars represent means ± SEM. (**B**) Quantitation of PrP^{PG14}-mCh aggregate colocalization with endogenous EEA1 (early endosomes), LAMP1 (endolysosomes) and Rab7 (LEs) (left). Representative immunofluorescence images of axons expressing PrP^{PG14}-mCh and stained with

antibodies to recognize endogenous EEA1 and Rab7 or LAMP1 and Rab7 (top right panels). Corresponding line scan plots for each channel (bottom right). Arrowheads indicate colocalization events. Scale bars = 10 µm. (**C**) Schematic diagram of a membrane flotation experiment (left) showing the 8/35 fraction ran on the Western blots in (C; right) and (D), and used as starting material for the Proteinase K (PK) and detergent insolubility assays in (E) and (F), respectively. Post-nuclear supernatant (PNS) from N2a cells homogenates were collected without detergent to prevent breaking of membranes and bottom loaded on a discrete sucrose gradient. Representative Western blots probed with an antibody against PrP (D13) show PrP^{PG14}-mCh signal (endogenous PrP shown in D), and against Rab7. Number of independent biological replicates = 3. (**D**) Representative Western blots showing PNS and 8/35 fractions from flotations of N2a cells transfected with PrP^{WT}- or PrP^{PG14}-mCh and probed with indicated antibodies. Number of independent replicates = 6. (**E**) Representative Western blots of PK-treated 8/35 membrane fractions of PrP^{WT} and PrP^{PG14} obtained from N2a cells as indicated in (**C**). Number of independent biological replicates = 3. (F) Representative Western blot of 8/35 membrane detergent soluble versus insoluble fractions obtained from PrP^{WT}-mCh and PrP^{PG14}-mCh expressing N2a cells as indicated in (**C**). Number of independent biological replicates = 2.

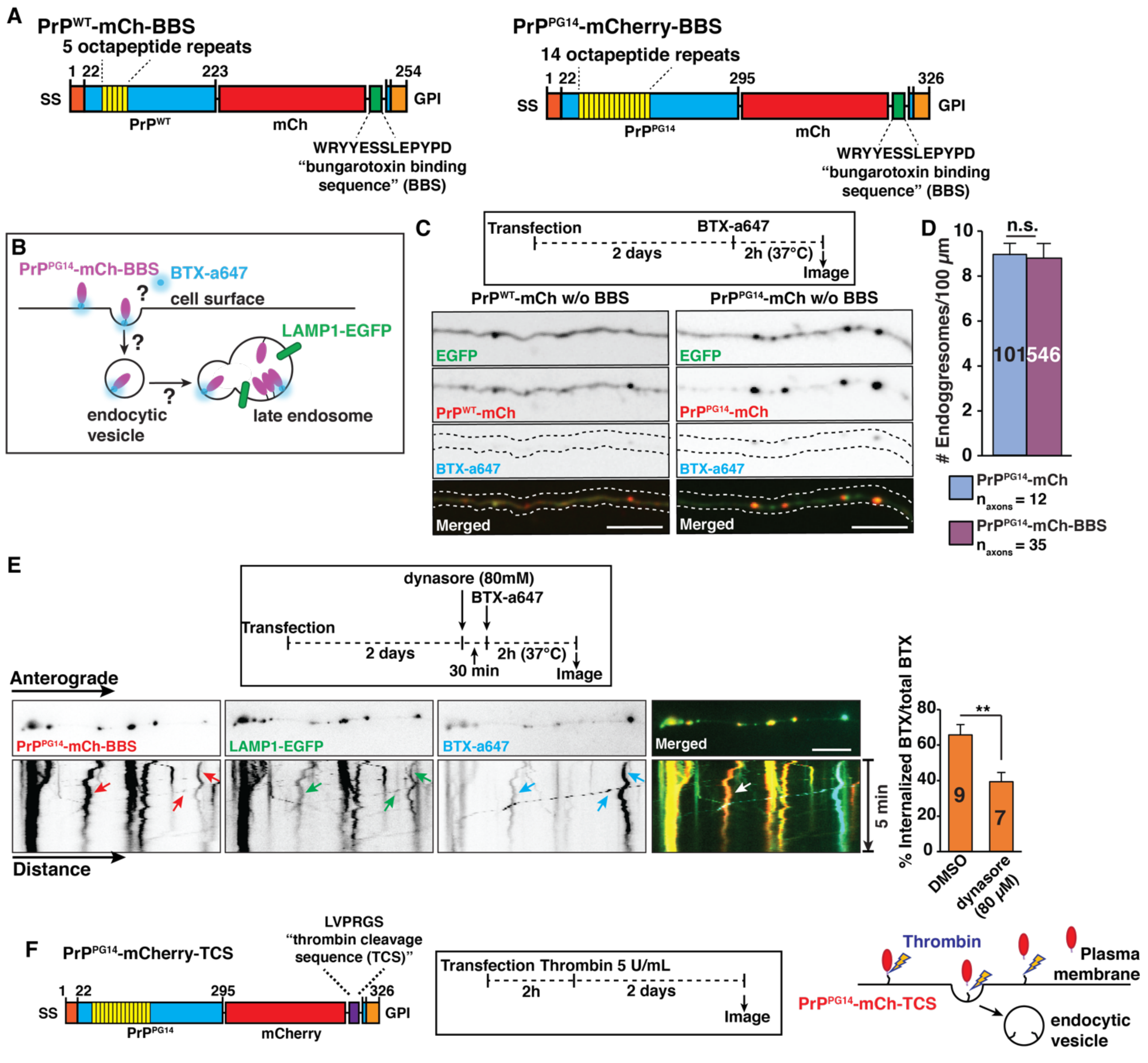

Fig. S4. PrP^{PG14} transiently traffics to the cell surface before intra-axonal aggregation. (**A**) Schematic of PrP^{WT}-mCh-BBS and PrP^{PG14}-mCh-BBS constructs. SS: signal sequence; GPI: GPI anchor. (**B**) Schematic of PrP^{PG14}-mCh-BBS internalization assay. (**C**) Experimental timeline of BTX-a647 labeling assay (top), and representative images of neurons expressing PrP^{WT}-mCh or PrP^{PG14}-mCh without BBS sequence, after treatment with BTX-a647 (bottom). Scale bars = 10 μm. (**D**) Quantitation of endoggresome densities from PrP^{PG14}-mCh and PrP^{PG14}-mCh-BBS-

expressing neurons treated with BTX-a647. Endoggresome numbers are shown inside bars. Bars represent means ± SEM. ns = not significant; Student's t-test. (**E**) Outline of Dynasore endocytosis assay (top). Representative first-frame images of time-lapse movie and kymographs of axons of neurons co-expressing PrP^{PG14}-mCherry-BBS, LAMP1-EGFP and treated with BTX-a647 (bottom left). Arrows point to cotransport. Scale bar = 10 μm. Quantitation of normalized percent internalized BTX-a647 signal (bottom right). Numbers of axons shown inside bars. Bars represent means ± SEM. **p<0.01, Student's t-test. (**F**) Schematic of PrP^{PG14}-mCh-TCS construct (left). SS: signal sequence; GPI: GPI anchor. Outline (middle) and schematic (right) of thrombin assay.

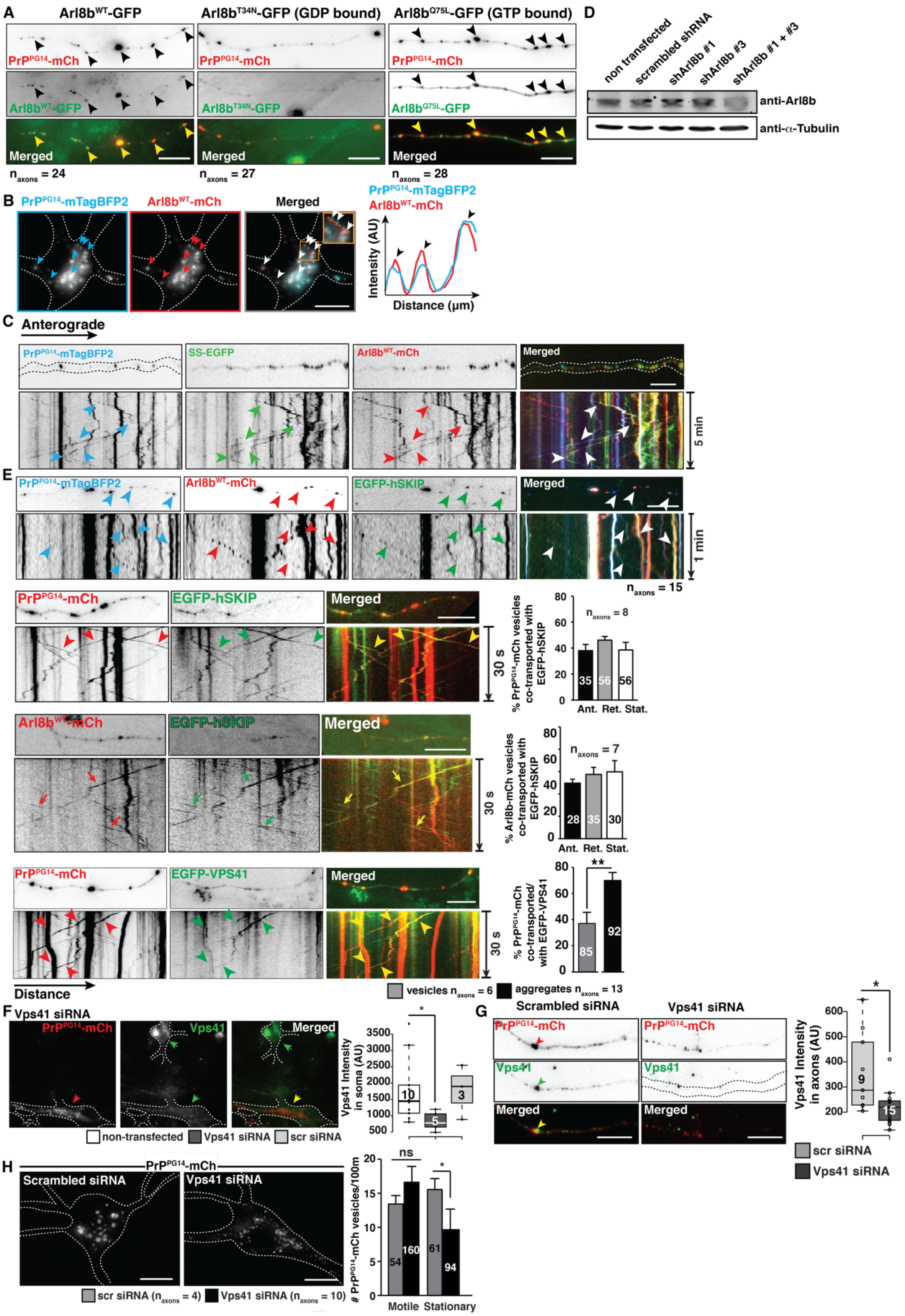
A
Arl8b^WT-GFP
Arl8b^T34N-GFP (GDP bound)
Arl8b^Q75L-GFP (GTP bound)
PrP^PG14-mCh
Arl8b^WT-GFP
Arl8b^T34N-GFP
Arl8b^Q75L-GFP
Merged
n_axons = 24
n_axons = 27
n_axons = 28
D
non transfected
scrambled shRNA
shArl8b #1
shArl8b #3
shArl8b #1 + #3
anti-Arl8b
anti-α-Tubulin
B
PrP^PG14-mTagBFP2
Arl8b^WT-mCh
Merged
Intensity (AU)
Distance (μm)
C
Anterograde
PrP^PG14-mTagBFP2
SS-EGFP
Arl8b^WT-mCh
Merged
5 min
E
PrP^PG14-mTagBFP2
Arl8b^WT-mCh
EGFP-hSKIP
Merged
1 min
n_axons = 15
PrP^PG14-mCh
EGFP-hSKIP
Merged
30 s
% PrP^PG14-mCh vesicles co-transported with EGFP-hSKIP
n_axons = 8
35
56
56
Ant. Ret. Stat.
Arl8b^WT-mCh
EGFP-hSKIP
Merged
30 s
% Arl8b-mCh vesicles co-transported with EGFP-hSKIP
n_axons = 7
28
35
30
Ant. Ret. Stat.
PrP^PG14-mCh
EGFP-VPS41
Merged
30 s
% PrP^PG14-mCh co-transported/ with EGFP-VPS41
**
85
92
Distance
vesicles n_axons = 6
aggregates n_axons = 13
F
Vps41 siRNA
PrP^PG14-mCh
Vps41
Merged
Vps41 Intensity in soma (AU)
*
10
5
3
non-transfected
Vps41 siRNA
scr siRNA
G
Scrambled siRNA
Vps41 siRNA
PrP^PG14-mCh
Vps41
Merged
Vps41 Intensity in axons (AU)
*
9
15
scr siRNA
Vps41 siRNA
H
PrP^PG14-mCh
Scrambled siRNA
Vps41 siRNA
scr siRNA (n_axons = 4)
Vps41 siRNA (n_axons = 10)
PrP^PG14-mCh vesicles/100m
ns
*
54
160
61
94
Motile
Stationary

Fig. S5. Arl8b effectors are recruited to PrP^{PG14} vesicles to promote fusion and aggregation. (**A**) Representative images of neurons co-expressing PrP^{PG14}-mCh and Arl8b^{WT}-GFP, Arl8b^{T34N}-GFP, or Arl8b^{Q75L}-GFP (left panels), and line scan intensity profiles of region indicated by dotted orange line inside inset (right). Arrowheads point to colocalization. Scale bar = 10 µm. (**B**) Representative images of hippocampal soma co-expressing PrP^{PG14}-mTagBFP and Arl8b^{WT}-mCh. Arrowheads point to colocalization. Scale bar = 10 µm. (**C**) Representative first-frame images of time-lapse movies, and corresponding kymographs of axons from neurons co-expressing PrP^{PG14}-mTagBFP2, SS-EGFP (from NPY), and Arl8b^{WT}-mCh. Arrowheads point to cotransport. Number of axons = 4. Scale bar = 10 µm. (**D**) Representative Western blot of N2a cell lysates treated with indicated shRNAs. Replicates = 3. (**E**) Representative first-frame images of time-lapse movies, and corresponding kymographs of axons from neurons co-expressing indicated constructs (left panels). Arrowheads point to cotransport. Numbers of axons for PrP^{PG14}-mTagBFP2, Arl8b^{WT}-mCh and EGFP-hSKIP = 15. Scale bars = 10 µm. Quantitation of cotransport/colocalization (right). Bars represent means ± SEM. Numbers of vesicles or endoggresomes are shown inside bars. (**F**) Immunofluorescence images of the soma of neurons expressing PrP^{PG14}-mCh, transfected with Vps41 siRNA, and stained with antibodies against Vps41 (left panels). Arrows point to two neurons either transfected or untransfected with Vps41 shRNA. Scale bar = 10 µm. Quantitation of Vps41 signal intensity (right). Boxes show lower (Q1) and upper (Q3) quartile and median. Whiskers mark the 9 to 91 percentile range. Datapoints outside of this range are represented by individual dots. Numbers of neurons are shown inside boxplots. (**G**) Representative axons from neurons co-transfected with PrP^{PG14}-mCh and Vps41 siRNA, stained with antibodies against Vps41 (left panels). Arrowheads point to endoggresomes. Scale bars = 10 µm. Quantitation of Vps41 signal intensity (right). Box shows

lower (Q1) and upper (Q3) quartile and median. Whiskers mark the 9 to 91 percentile range. Datapoints outside of this range are represented by individual dots. Numbers of neurons are shown inside boxplots. (**H**) Representative images of soma of neurons co-transfected with PrP^{PG14}-mCh and shRNA scrambled or Vps41 siRNA (left panels). Scale bars = 10 μm. Quantitation of motile and stationary axonal PrP^{PG14}-mCh vesicle densities in neurons co-transfected with scrambled or Vps41 shRNAs (right). Numbers of vesicles are shown inside bars.

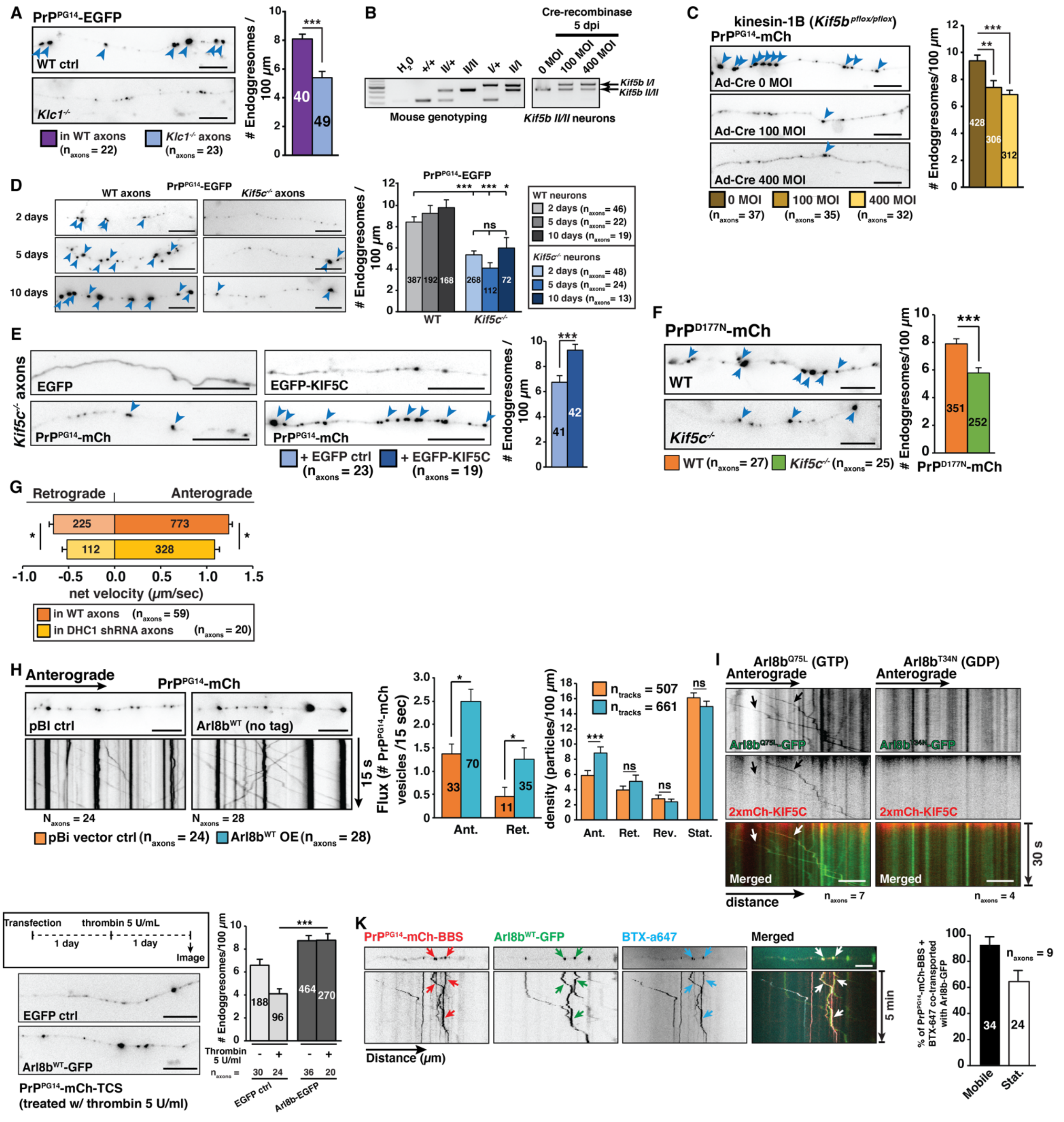

Fig. S6. ARESTA drives endoggresome formation in axons. (**A**) Images of WT and *Klc1*$^{-/-}$ axons expressing PrP^{PG14}-EGFP (left). Arrowheads point to endoggresomes. Scale bar = 10 µm. Quantitation of endoggresome densities (right). Bars represent means ± SEM. Numbers of

endoggresomes are shown inside bars. ***p<0.001, Student's t-test and Kruskal- Wallis test. (**B**) DNA gel of genotyping bands from Kinesin-1B neuron extracts treated with increasing amounts of cre-recombinase adenovirus (AVV-cre) at 5 days post-infection (dpi). Excision of *Kif5B* II occurs following cre-treatment to convert to a kinesin-1B I/+ product. MOI = Multiplicity of infection. (**C**) Images of *Kif5B* conditional knockout axons expressing PrP^{PG14}-mCh and treated with indicated AAV-cre units (left). Arrowheads point to endoggresomes. Scale bar = 10 μm. Quantitation of endoggresome densities (right). Bars represent means ± SEM. Numbers of endoggresomes are shown inside bars. **p<0.01, ***p<0.001, Student's t-test and Kruskal-Wallis test. (**D**) Images of axons from WT and *Kif5c*$^{-/-}$ neurons expressing PrP^{PG14}-EGFP at various time points post-transfection (left). Arrowheads point to endoggresomes. Scale bars = 10 μm. Quantitation of endoggresome densities (right). (**E**) Images of *Kif5c*$^{-/-}$ axons co-expressing soluble EGFP or EGFP-KIF5C and PrP^{PG14}-mCh (left). Scale bar = 10 μm. Quantitation of endoggresome densities (right). Bars represent means ± SEM. Numbers of endoggresomes are shown inside bars. ***p<0.001, Student's t-test and Kruskal- Wallis test. (**F**) Representative images of PrP^{D177N}-mCh aggregates in WT and *Kif5C*$^{-/-}$ axons (left). Arrowheads point to endoggresomes. Scale bar = 10 μm. Quantitation of aggregate densities (right). Bars represent means ± SEM. Numbers of endoggresomes are shown inside bars. ***p<0.001, Student's t-test and Kruskal- Wallis test. (**G**) Average net axonal velocities of PrP^{PG14}-EGFP vesicles in WT and DHC1 shRNA neurons. Numbers of tracks are shown inside bars. Bars represent means ± SEM. *p<0.05, Kruskal- Wallis test. (**H**) Representative first-frame images of time-lapse movies, and corresponding kymographs of axons from neurons expressing PrP^{PG14}-mCh and transfected with empty control pBi vector or untagged $Arl8b^{WT}$ (left panels). Scale bars = 10 μm. Quantitation of PrP^{PG14}-EGFP vesicle (middle). Quantitation of vesicle densities (right). Numbers of vesicles are

shown inside bars. Ant. = anterograde; Ret. = retrograde; Rev. = reversal; Stat. = stationary. Bars represent means ± SEM. *$p<0.05$, ***$p<0.001$, ns = not significant, Kruskal- Wallis test. (**I**) Representative kymographs of axons co-expressing Arl8b^{Q75L}-GFP or Arl8b^{T34N}-GDP and 2xmCh-KIF5C. Arrows indicate cotransport. Scale bars = 10 μm. (**J**) Outline of thrombin assay (top left), and representative images of axons of neurons co-expressing PrP^{PG14}-mCh-TCS and soluble EGFP or Arl8b^{WT}-GFP (bottom left). Scale bar = 10 μm. Quantitation of endoggresome densities (right). Number of endoggresomes are shown inside bars. Bars represent means ± SEM. ***$p<0.001$, Kruskal- Wallis test. (**K**) Representative first frames of time-lapse movie, and kymographs of axons expressing PrP^{PG14}-mCh-BBS, Arl8b^{WT}-GFP, and labeled with BTX-a647 (left panels). Arrows point to cotransport. Scale bar = 10 μm. Quantitation of cotransport (right). Numbers of vesicles are shown inside bars. Bars represent means ± SEM.

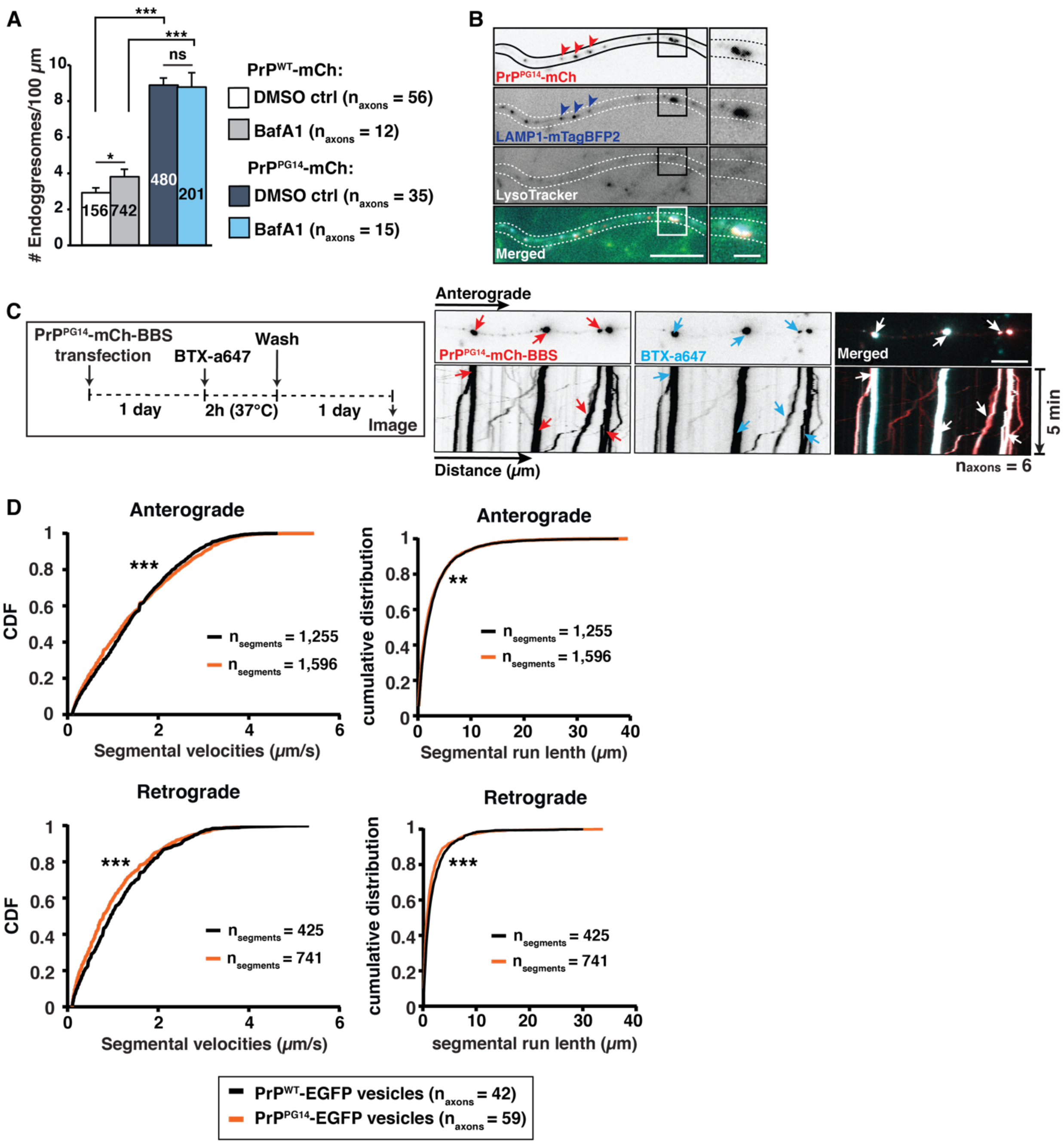

Fig. S7. Lysosomal degradation and retrograde transport deficits in PrP^{PG14}-expressing axons. (**A**) Quantitation of axonal PrP^{PG14}-mCh endoggresome densities in neurons treated with BafA1. Numbers of endoggresomes are shown inside bars. Bars represent means ± SEM**.** ***p<0.001, Kruskal- Wallis test. (**B**) Representative images of axons of neurons co-expressing

PrP^{PG14}-mCh and LAMP1-mTagBFP2 and treated with LysoTracker (left panels). Arrowheads point to colocalization. Scale bar = 10 μm. Enlargements of boxed regions (right panels). Scale bar = 2.5 μm. Number of axons = 21. (**C**) Outline of BTX assay (left). Representative first frames of time-lapse movie, and kymographs of BTX-a647-labeled PrP^{PG14}-mCh-BBS axons (right). Arrows point to cotransport. Scale bar = 10 μm. (**D**) Cumulative distribution frequencies (CDFs) of anterograde (top) and retrograde (bottom) segmental velocities and segmental run length of PrP^{WT}- and PrP^{PG14}-EGFP vesicles. $^{**}p<0.01$, $^{***}p<0.001$, Kolmogorov-Smirnov test.

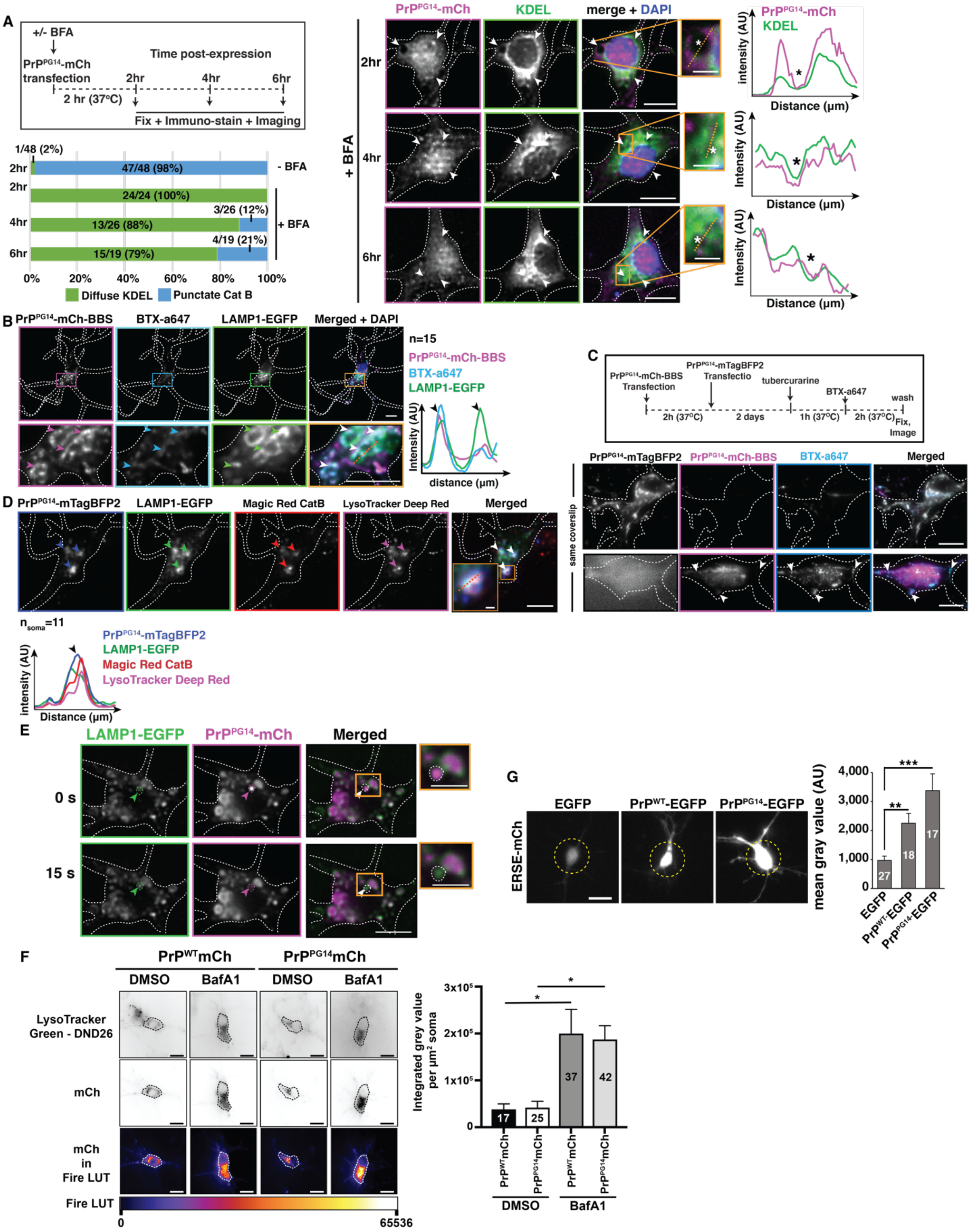
A
+/- BFA
PrP^PG14-mCh transfection
Time post-expression
2hr
4hr
6hr
2 hr (37°C)
Fix + Immuno-stain + Imaging
1/48 (2%)
47/48 (98%)
- BFA
24/24 (100%)
3/26 (12%)
13/26 (88%)
4/19 (21%)
15/19 (79%)
+ BFA
0%
20%
40%
60%
80%
100%
Diffuse KDEL
Punctate Cat B
PrP^PG14-mCh
KDEL
merge + DAPI
+ BFA
Intensity (AU)
Distance (µm)
B
PrP^PG14-mCh-BBS
BTX-a647
LAMP1-EGFP
Merged + DAPI
n=15
distance (µm)
C
PrP^PG14-mCh-BBS Transfection
PrP^PG14-mTagBFP2 Transfectio
tubercurarine
BTX-a647
wash
2h (37°C)
2 days
1h (37°C)
Fix, Image
PrP^PG14-mTagBFP2
Merged
same coverslip
D
Magic Red CatB
LysoTracker Deep Red
n_soma=11
intensity (AU)
E
0 s
15 s
F
PrP^WTmCh
PrP^PG14mCh
DMSO
BafA1
LysoTracker Green - DND26
mCh
mCh in Fire LUT
Fire LUT
0
65536
Integrated grey value per µm² soma
3x10^5
2x10^5
1x10^5
17
25
37
42
G
EGFP
PrP^WT-EGFP
PrP^PG14-EGFP
ERSE-mCh
mean gray value (AU)
4,000
3,000
2,000
1,000
27
18
17

Fig. S8. Somatic lysosomal clearance of neuronal PrP^{PG14}. (**A**) Outline of BFA assay (top left), and quantification of PrP^{PG14}-mCh colocalization with KDEL or CatB (bottom left). Numbers inside/above bars are total number and percentage of observed neurons. Representative images of soma of neurons expressing PrP^{PG14}-mTagBFP2 and stained with antibodies against ER (KDEL) or with DAPI nuclear marker at indicated time points in the presence of BFA. Arrowheads and asterisks in insets point to colocalization or non-colocalization events, respectively, also shown in enlarged insets and line scan intensity profiles of dotted lines (right). Scale bars = 10 μm. Scale bars of insets = 250 nm. (**B**) Representative images of a neuronal soma co-transfected with PrP^{PG14}-mCh-BBS and LAMP1-EGFP, and labeled with BTX-a647 (top left panels), and enlargement of insets (bottom left panels). Scale bars = 10 μm. Line scan intensity profiles of dotted lines (right). (**C**) Outline of BBS internalization assay (top). Representative images of two neurons taken from the same coverslip, sequentially transfected with PrP^{PG14}-mCh-BBS and PrP^{PG14}-mTagBFP2 constructs. Scale bars = 10 μm. (**D**) Representative images of the soma of a neuron co-transfected with PrP^{PG14}-mTagBFP2 and LAMP1-EGFP, and treated with Magic Red and LysoTracker Deep Red (top panels). Arrowheads and asterisk point to colocalization or non-colocalization events, respectively. Scale bar of main panels and of inset = 10 μm and 1 μm, respectively. Enlarged insets and line scan intensity profiles (bottom). (**E**) Images of a neuron co-expressing LAMP1-EGFP and PrP^{PG14}-mCh at two time points. Scale bar = 10 μm. Arrowheads point to a PrP^{PG14}-mCh disappearing vesicle (enlarged in inset). Scale bars in insets = 5 μm. (**F**) Representative black/white inverted fluorescence images of the soma of DMSO or BafA1- and LysoTracker Green-treated PrP^{WT}-mCh and PrP^{PG14}-mCh neurons (left panels). BafA1 treatment was for 12 hrs. Fire Look-up table (LUT) is shown on the bottom panels. Scale bar = 20 μm. Number of independent biological

replicates = 3. Quantitation of integrated grey values of soma data on left panels (right). Calculation of intensities was done on regions of interest (ROIs) drawn around soma on the mCh channel, and grey values are represented as integrated/cumulative grey value per μm^2 of soma. Numbers of soma are indicated inside bars. Bars represent means ± SEM**.** *p<0.05, Student's t-test. (**G**) Representative images (left panels) and quantitation of intensity levels (right) in neurons co-transfected with ERSE-mCh and EGFP (control), PrP^{WT}-EGFP, or PrP^{PG14}-EGFP. Dotted lines indicate 30 μm diameter circles used to measure total gray intensity values. Scale bar = 20 μm. Bars represent means ± SEM**.** **p<0.01, ***p<0.001, Student's t-test. Numbers of neurons are shown inside bars.

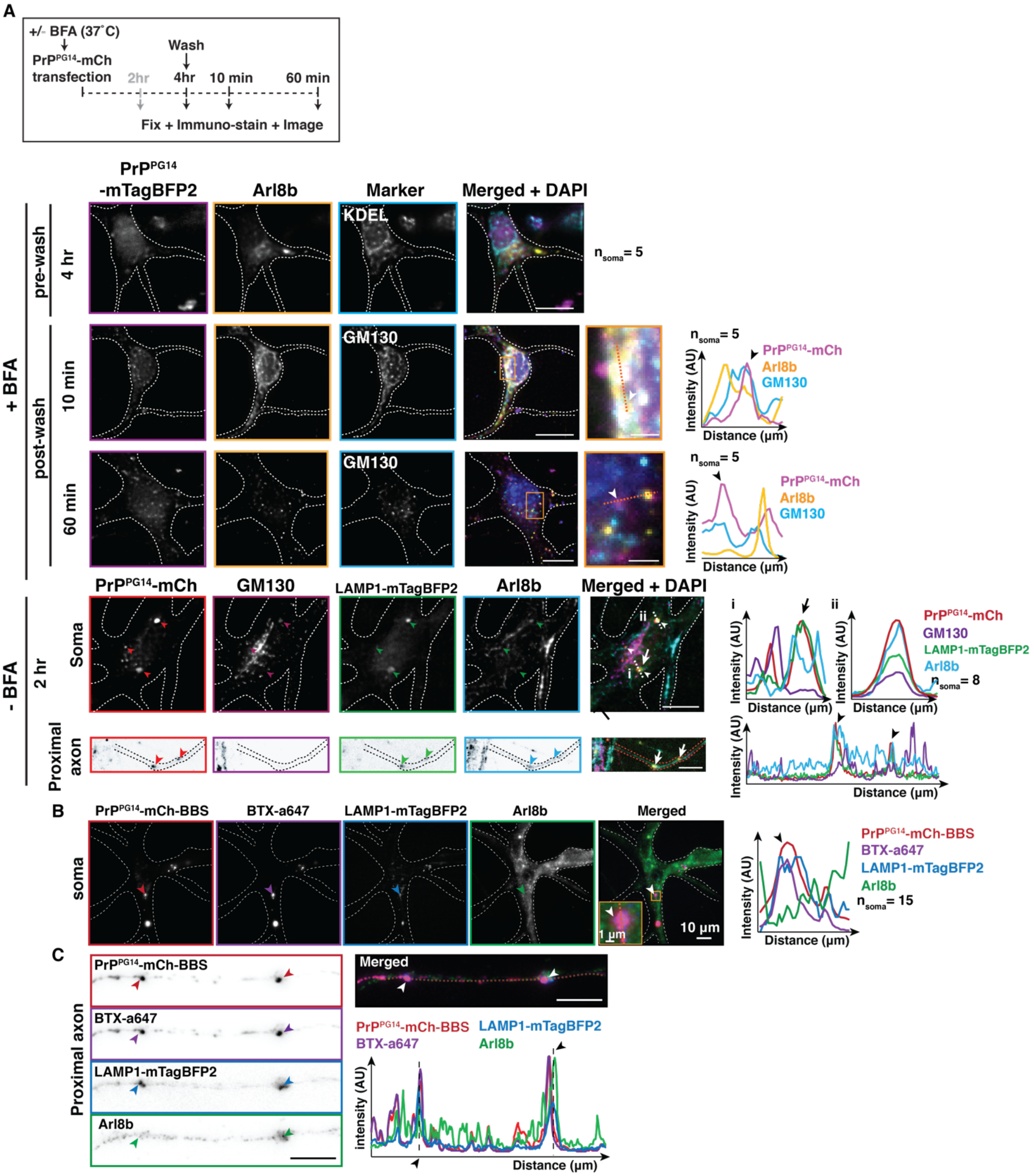

Fig. S9. Arl8b is a key determinant of PrP^{PG14} axonal entry. (**A**) Outline of BFA assay (top). Representative images of soma and proximal axons of different neurons expressing PrP^{PG14}-mTagBFP2 or PrP^{PG14}-mCh + LAMP1-mTagBFP2 and stained with antibodies against Arl8b,

ER (KDEL), Golgi (GM130), or with DAPI nuclear marker at indicated time points and conditions, with or without BFA treatment (bottom panels). Graphs to the right are of line scans of dotted lines inside insets. Arrowheads point to colocalization events, also shown in enlarged insets and in line scan intensity profiles. Scale bars in main panels = 10 μm. Scale bars in insets = 500 nm. (**B**) Representative images of PrP^{PG14}-mCh-BBS internalization in the soma (left panels) of neurons co-transfected with PrP^{PG14}-mCh-BBS and LAMP1-mTagBFP2, labeled with BTX-a647, and stained with an antibody against Arl8b. Arrowheads point to colocalization events, also shown in enlarged insets and in line scan intensity profile (right). Scale bars in main panel = 10 μm. Scale bars in inset = 1 μm. (**C**) Representative images of PrP^{PG14}-mCh-BBS internalization in axons (left and top right panels) of neurons co-transfected with PrP^{PG14}-mCh-BBS and LAMP1-mTagBFP2, labeled with BTX-a647, and stained with an antibody against Arl8b. Arrowheads point to colocalizing endoggresomes. Line scan intensity profile (right). Scale bar = 10 μm.

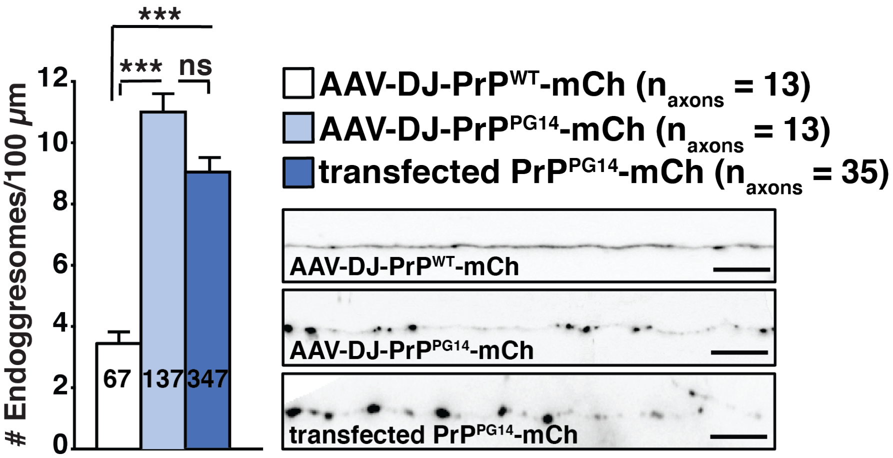

Fig. S10. Expression of AAV-DJ-PrP^{PG14}-mCh results in the formation of endoggresomes. Quantitation of endoggresome densities (left) and representative images (right) of axons from neurons transduced with AAV-DJ-PrP^{PG14}-mCh. Numbers of endoggresomes are shown inside bars. Scale bar = 10 μm.

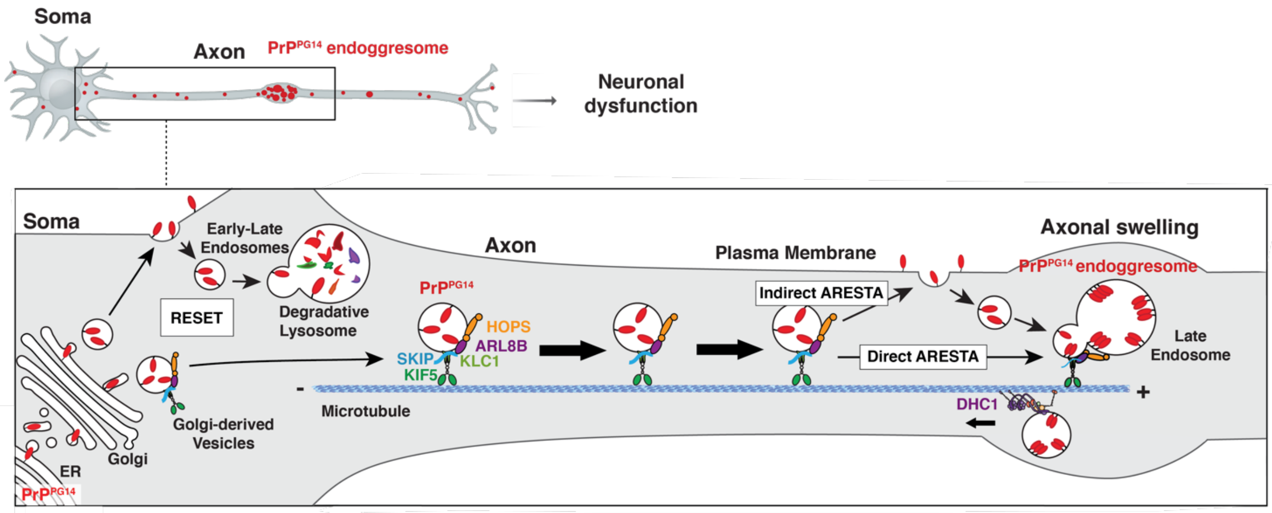

Fig. S11. Neuronal endolysosomal trafficking pathways that clear misfolded PrP in the soma and promote endoggresome formation in axons. Model showing endosomal pathways governing mutant PrP degradation versus aggregation in neurons. RESET acts in the soma to degrade PrP^{PG14} via the cell surface. Arl8b/kinesin-1/SKIP/HOPS earmarks PrP^{PG14} vesicles as endosomes and drives their axonal entry and toward ARESTA-dependent aggregation. Aggregation occurs following indirect targeting of PrP^{PG14} to late endosomes via the axonal cell surface, via its direct homotypic fusion, or both. Dynein-mediated retrograde transport and axonal degradative capacity are impaired in axons thus promoting the maintenance of endoggresomes in axons.

Movie S1.

Axonal transport of PrP^{PG14}-mCh vesicles and aggregates. Inverted contrast movie showing axonal transport of PrP^{PG14}-mCh vesicles and aggregates in an axon of a cultured hippocampal neuron.

Movie S2.

Axonal transport of PrP^{WT}-mCh vesicles. Inverted contrast movie showing axonal transport of PrP^{WT}-mCh vesicles in an axon of a cultured hippocampal neuron.

Movie S3.

Degradation of PrP^{PG14}-mCh in lysosomes in the soma. Movie of a neuron co-expressing LAMP1-EGFP (green) and PrP^{PG14}-mCh (red) showing PrP^{PG14}-mCh puncta disappearing.
